# Advances in Image-Guided Ablation Therapies for Solid Tumors

**DOI:** 10.3390/cancers16142560

**Published:** 2024-07-17

**Authors:** Warren A. Campbell, Mina S. Makary

**Affiliations:** 1 Division of Vascular and Interventional Radiology, Department of Radiology, University of Virginia, Charlottesville, VA 22903, USA; 2 Division of Vascular and Interventional Radiology, Department of Radiology, The Ohio State University Wexner Medical Center, Columbus, OH 43210, USA

**Keywords:** interventional radiology, interventional oncology, image-guided ablation therapies, solid tumor ablation

## Abstract

**Simple Summary:**

Image-guided solid tumor ablation therapy is an expanding practice designed to effectively target and eradicate malignant tissue through noninvasive or minimally invasive techniques. There exist several ablation systems that can utilize localized heating or mechanical stress to induce cell death of malignant cells. The provider selects a technique based on patient comorbidities, the type of malignancy, the size and location of the tumor, and the desired goal of therapy. Over the past two decades, several studies have validated the safety and efficacy of these techniques in humans to be used as an alternative to medical and surgical therapy or as an adjunct in a hybrid treatment plan. Compared to conventional surgical therapies, these procedures are better tolerated, with reduced morbidity and mortality, providing options to patients that are not surgical candidates. This article reviews the new and common techniques in solid tumor ablation and the research supporting their use in oncological treatment.

**Abstract:**

Image-guided solid tumor ablation methods have significantly advanced in their capability to target primary and metastatic tumors. These techniques involve noninvasive or percutaneous insertion of applicators to induce thermal, electrochemical, or mechanical stress on malignant tissue to cause tissue destruction and apoptosis of the tumor margins. Ablation offers substantially lower risks compared to traditional methods. Benefits include shorter recovery periods, reduced bleeding, and greater preservation of organ parenchyma compared to surgical intervention. Due to the reduced morbidity and mortality, image-guided tumor ablation offers new opportunities for treatment in cancer patients who are not candidates for resection. Currently, image-guided ablation techniques are utilized for treating primary and metastatic tumors in various organs with both curative and palliative intent, including the liver, pancreas, kidneys, thyroid, parathyroid, prostate, lung, breast, bone, and soft tissue. The invention of new equipment and techniques is expanding the criteria of eligible patients for therapy, as now larger and more high-risk tumors near critical structures can be ablated. This article provides an overview of the different imaging modalities, noninvasive, and percutaneous ablation techniques available and discusses their applications and associated complications across various organs.

## 1. Introduction

The discovery that electromagnetic waves could pass through tissue and cause heating led to the invention of electrocautery [1]. Localized ablation as a technique evolved from the surgical implementation of the Bovie knife. Researchers soon discovered that increasing the intensity would effectively “char” the tissue, leading to the early form of radiofrequency thermal ablation. The prevalence of localized ablation techniques increased over time with the advancement of the laparoscopic technique and introduction of minimally invasive surgery in the 1970s. With the concurrent development of CT scanners and improvements to ultrasound (US) imaging, image-guided procedures were being developed for biopsy and brachytherapy [2,3]. By the late 1990s, there was a robust expansion in the type of imaging and ablation modality being actively researched.

In the multimodal, interdisciplinary approach in the treatment of cancer, image-guided percutaneous ablation therapies of solid tumors became an important interventional-oncology-driven therapy that has a growing role in cancer therapies. The future development of these therapies is to improve precision for targeted apoptosis while preserving the surrounding organ parenchyma. This is being accomplished by improved visualization with real-time and hybrid imaging, where imaging modalities can be combined to compensate for specific limitations. When using thermal-based techniques, the thermodynamics of temperature change significantly impacts the distribution of ablation. Therefore, improved temperature monitoring or intelligent responsive systems controlling the parameters can ensure complete tumor targeting without expanding into healthy parenchyma. Clinical research is focused on testing these tools to improve patient survival and reduce complications.

This review will examine clinical and technical advancements of well-studied effective ablation therapies: radiofrequency ablation (RFA), microwave ablation (MWA), cryoablation, irreversible electroporation (IRE), high-intensity focused ultrasound (HIFU), and histotripsy.

## 2. Image-Guided Ablation

Image-guided percutaneous ablation is characterized by the ability to target an applicator to tumors percutaneously with image guidance [4]. Ultrasound is the most cost-effective and abundantly available imaging modality that provides real-time feedback without exposure to ionizing radiation. The primary limiting factors are the sensitivities for deep and small masses, gas-filled structures, and large body habitus. Some of these factors can be negated by the addition of microbubble contrast agent (contrast-enhanced ultrasound, CEUS), which improves the echogenicity of an image. This can only be applied to one 2D cross-sectional image at a time but can be performed on several images with a single vial of contrast. Despite the improvement in the dynamic range of the image, this often cannot overcome some of the aforementioned limitations.

Computed tomography (CT) imaging offers a more detailed wide field of view for probe targeting that can visualize important and obstructing anatomy. While detailed, CT scans offer only a snapshot of the current anatomy. Cone beam CT (CBCT) evolved for volumetric 3D reconstruction from 2D XR images. For interventions, it allows improved visualization and feedback to the operator. Additional benefits include less radiation exposure and super-imposing the imaging on live fluoroscopy for continued targeting guidance [5].

Magnetic resonance imaging (MRI) offers the advantages of real-time imaging and greater soft-tissue resolution. This is particularly beneficial for thermal sensing to evaluate the extent of procedural ablation. Based on the approach, the imaging mechanism selected complements the limitation of the technique (Table 1).

An evolving field of research is using a combination of these imaging techniques to compensate for their individual limitations, also known as hybrid or fusion imaging. From an interventional perspective, the ability to target a mass is facilitated by real-time feedback. While US offers the most flexibility, there is low resolution and limited tissue depth. US combined with CT or MRI opens the opportunity to target US inconspicuous masses [6]. Additionally, some masses may only be well delineated from a 18F-fluorodeoxyglucose positron emission tomography (PET) signature, which can be fused with US and CT for proper probe placement [7].

Furthermore, new nanoparticle contrast agents are being researched to improve tissue resolution. These macromolecules contain unique properties that will improve tissue contrast and spatial resolution to CT and MRI to the micron or nanometer scale [8]. These agents consist of polymers, liposomes, beads, bubbles, and nanoparticles. Utilizing the combination of contrast agents and imaging modalities, effective targeting of small and poorly distinguishable masses has become possible and broadened the range of potential candidates for ablation.

## 3. Ablation Techniques

Ablation techniques are generally categorized into thermal and non-thermal mechanisms. Radiofrequency ablation (RFA), microwave ablation (MWA), and cryoablation rely on the conversion of energy from the probe to cause thermal stress and subsequent cell death. Non-thermal ablation modalities are heterogenous, relying on either electrical stimulation in irreversible electroporation (IRE), or mechanical destruction of tissue through high-intensity focused ultrasound (HIFU) or histotripsy. The biomechanics of tumor targeting impact the advantages and disadvantages of each technique when implemented in a clinical setting (Table 2). We will review the mechanism, clinical technique, and outcomes targeting solid tumors [9].

## 4. Radiofrequency Ablation

### 4.1. Mechanism and Technique

Thermal ablation is a well-studied technique where temperatures >60 degrees Celsius are known to cause coagulation necrosis of the target tissue. The most common way to accomplish this is with radiofrequency ablation (RFA), where the resistance to the fluctuating electrical current begins heating the tissue proximal to the probe and extends via thermal conduction to heat the surrounding tissue. 

RF energy stands out as the most extensively researched and clinically applicable percutaneous ablation method. In RF ablation, electrical current oscillates between electrodes via ion channels present in most biological tissues [10]. This technique implements a basic electrical circuit, with the generator, cabling, electrodes, and tissue acting as the resistive elements within the current loop. Heating is most pronounced in regions with high current density, primarily affecting tissues closest to the electrode, while peripheral areas receive heat through thermal conduction. 

Ablative heating induces tissue dehydration and water vaporization, leading to significant increases in circuit impedance. These abrupt impedance changes can serve as feedback signals in RF generators. To counteract impedance-related hindrances to current flow, various methods can be employed, such as expanding the electrode surface area, pulsing input power, or saline injections. 

Electrodes play a crucial role in thermal ablation, with each generator’s power output and control algorithms tailored to its specific electrodes [11,12]. Presently, most RF ablation systems operate in a monopolar mode, utilizing two types of electrodes: interstitial electrodes for delivering energy to the tumor, generating high current density and localized heating, and dispersive electrodes (or ground pads) placed on the skin surface to disperse energy over a larger area, minimizing the risk of skin damage. 

Monopolar electrodes include straight insulated needles with an exposed metallic tip and multitined designs for spatial energy distribution, reducing tissue charring and enabling higher power deposition [13]. Internally cooled electrodes circulate fluid within the active tip to maintain lower interface temperatures, enhancing energy delivery. Multiple tines extending from a single sheath aim to improve heating efficiency and create larger ablation zones. Bipolar systems use electrical currents between two interstitial electrodes, eliminating the need for a ground pad and focusing heating between the electrodes. To enhance energy delivery, bipolar systems may use saline infusion, while multipolar operation alternates between pairs of bipolar electrodes for more effective ablation.

Thermal ablation techniques, including RFA, are limited in their ability to target tumors near large blood vessels, as this heat sink negates the necessary ablation margins. One strategy to overcome this barrier is to design electrodes that improve energy delivery to the mass quickly. These strategies include bipolar electrodes and multitined expandable electrodes [14]. Internally cooled electrodes have shown success by preventing the proximal tissue from becoming overheated and charred, reducing heating to the tumor margins [15,16]. Alternatively, large vessels can be occluded to reduce arterial flow to the affected tissue. Studies have validated that the balloon occlusion of the hepatic artery reduces the heat sink effect without significant increases in morbidity or mortality.

A focus of RFA development (and other thermal ablation methods) has been on increasing the sensitivity and accuracy of temperature monitoring. Conventional temperature monitoring techniques include fiber optics and thermocouples. These are fast and accurate, but at the cost of being invasive with lower spatial resolution. Magnetic resonance thermal imaging (MRTI) provides high resolution of the tissue and temperature gradings. Of the various weighting methods, the proton resonance frequency shift (PRFS) method is often preferred due to his wide range of accuracy [17]. Some developments have been made in using spectral CT to perform temperature measurements based on the principle of temperature-dependent changes in tissue density [18]. This can be performed effectively for cryoablation, where an ice ball is clearly detected but does not accurately measure cytotoxic near-freezing temperatures.

### 4.2. Hepatic Ablation

RFA stands out as a primary ablative method for hepatocellular carcinomas (HCCs) smaller than 20 mm. Various studies have assessed RFA’s effectiveness compared to resection and have confirmed it as a noninvasive and efficient ablative therapy [19,20,21,22]. A meta-analysis of the literature of five retrospective studies regarding the survival of RFA in hepatocellular carcinoma showed comparable one-, three-, and five-year patient survival ranging from 33% to 55%. When compared to redo hepatic resection, there was no significant difference in overall survival (OS) outcomes, but with reduced morbidity relative to redo hepatic resections [22,23]. For small, single hepatocellular carcinomas, RFA can show superior one-, three-, and five-year overall and disease-free survival (DFS) [24]. Park et al. conducted a similar retrospective study comparing curative RFA with hepatic resection [25]. This study found similarities between RFA and hepatic resection in morbidity, mortality, and OS. There was a marginal increase in DFS, suggesting that patients that are good surgical candidates should consider resection in tumors < 5 cm or three or fewer nodules < 3 cm. 

Kim et al. performed a retrospective study of 604 patients, reviewing OS and DFS over 5 and 10 years. Patients that underwent RFA had 5- and 10-year OS of 82.1% and 61.2%, which were similar for hepatic resection [26]. Hepatic resection was more effective than RFA concerning DFS, at 60.6% vs. 39.4% and 37.5% vs. 25.1%, respectively. This study reaffirmed that patients that can tolerate a hepatectomy with lower risk of morbidity and mortality will have a higher curative rate. However, RFA maintains similar outcomes with OS with lower risks for procedural morbidity and mortality. When offered as a palliative measure to reduce tumor burden, RFA continues to offer utility in the management of hepatocellular carcinoma.

Current advancements in the research of hepatic RFA ablation focus on the efficacy of using RFA with combination therapies, such as chemotherapy, radiation therapy, and transhepatic arterial chemoembolization [22]. While there are variable data regarding the efficacy of combination therapy, RFA continues to be a well-researched and validated tool in the treatment of solid hepatic masses. 

### 4.3. Pancreatic Ablation

While surgical resection stands as the sole curative option, it remains accessible to only a minority of patients. Although RFA is a recognized approach in managing solid organ tumors, its efficacy in pancreatic cancer treatment is still controversial. Over the past decade, there has been a growing adoption of RFA due to its potential as a viable palliative measure for unresectable pancreatic cancer. RFA achieves tumor cytoreduction through various mechanisms, including coagulative necrosis, protein denaturation, and stimulation of anti-cancer immune responses. Despite controversies surrounding its safety profile due to a notable risk of complications, limited prospective and retrospective studies have demonstrated promising outcomes regarding its use in palliative care for unresectable pancreatic malignancies [27]. 

RFA for pancreatic tumors can be executed through various methods, including intraoperative, percutaneous guided by ultrasound or radiologic imaging, and endoscopic approaches utilizing endoscopic ultrasound (EUS) or endoscopic retrograde cholangiopancreatography (ERCP) [28,29]. RFA heat sink effects can impact the effective margins of the ablation zone, increasing the risk of damaging the bowel, biliary tract, and intraparenchymal pancreatic structures and endocrine cells [30]. In preliminary feasibility studies, the complications included acute pancreatitis, pancreatic fistula, gastrointestinal hemorrhage, sepsis, portal vein thrombosis, and structural damage to the duodenum or bile duct [31]. Efforts to minimize these complications focus on controlling the spread of thermal damage, with active cooling of the duodenum and inferior vena cava during ablation.

Compared to prior studies that performed RFA in conjunction with laparotomy for visualization and access, D’Onofrio et al. performed percutaneous RFA of biopsy-confirmed, unresectable, locally advanced pancreatic adenocarcinoma in 18 patients, with a technical success rate of 93% [32]. While initially effective at cytoreduction, 44.4% of patients had increased tumor growth in one month. Mizandari et al. conducted a similar study on 134 patients in addition to stent placement for malignant bile and pancreatic duct obstructions, with a technical success rate of 97% [33]. Long-term stent patency and tumor size were not followed in this study, but the immediate post-operative period was well tolerated by patients, with two experiencing contrast extravasation managed by stent placement. These patients had no evidence of hemorrhage, vessel injury, visceral injury, or infection.

### 4.4. Renal Ablation

Ablative methods have emerged as alternatives to partial nephrectomy, particularly for patients with significant comorbidities and heightened risks of anesthesia-related complications. These techniques offer advantages, such as outpatient procedures without general anesthesia, thereby reducing the potential for complications. The ideal location for kidney tumor ablation is a noncentral area of the kidney where the tumor is less than 4 cm in diameter. The least challenging location is posterior exophytic, while the most challenging is central hilar. Tumors in the central or hilar regions are more difficult to treat and have a higher risk of incomplete treatment or recurrence [34].

Green et al. investigated the intermediate-term outcomes post-ablation, with most patients experiencing almost complete resolution on CT imaging. The mean age of patients undergoing ablation was 63.35 years, with the majority diagnosed with clear-cell RCC on histology. Renal function remained stable pre- and post-ablation in our study. However, other studies have reported renal function deterioration in some cases, particularly among those with pre-existing renal impairment [35]. 

Complications following renal ablation are relatively rare, with studies reporting overall complication rates ranging from 8% to 13%. Recurrence rates post-ablation vary, with our study showing recurrence in 21.4% of cases over three years of follow-up, primarily in clear-cell carcinoma cases. Tumor size, gender, RCC type, and lesion location do not significantly correlate with recurrence [36]. 

In summary, renal ablation techniques offer promising outcomes for managing small kidney masses, with low complication rates and favorable intermediate-term results. However, further studies are needed to better understand predictors of recurrence and long-term oncologic outcomes.

### 4.5. Lung Ablation

Research following the outcomes of patients with solid lung tumors differentiates primary and metastatic lesions. For those with primary lung tumors, patients treated with RFA for early-stage non-small-cell lung cancer (NSCLC) were those unable or unwilling to undergo surgery, and those who may benefit more from RFA. Additionally, candidates are limited by anatomic criteria. For curative purposes, lesions larger than 5 cm are excluded from RFA, and those between 3 and 5 cm are approached cautiously due to a high recurrence rate. Additionally, nodules located within 1 cm of the trachea, main bronchi, esophagus, and central vessels are excluded from RFA due to the substantial risk of complications and difficulty in achieving complete ablation [37].

Video-assisted thoracoscopic surgery (VATS) has traditionally been the gold standard for NSCLC treatment, but factors such as aging populations, increased comorbidities, and low cardiac or lung function in some patients may necessitate alternative options, such as RFA or high-dose radiotherapy. Studies have shown that RFA can be a viable option for patients with poor physical conditions, as it has minimal impact on lung function. Guidelines recommend considering RFA for patients with NSCLC who are intolerant or refuse surgery or radiotherapy [38]. 

Furthermore, RFA feasibility should be carefully evaluated in patients with multiple primary ground-glass opacities (GGOs), which are increasingly detected due to advancements in CT imaging. While surgical treatment for multiple GGOs is complex, studies suggest favorable outcomes with RFA for nodules with a high GGO component. However, longer-term follow-up and larger clinical cohorts are needed to fully assess the efficacy of RFA in these cases. 

For treatment of metastatic disease, various studies have followed patient survival and disease progression. Among the studies assessing various ablation techniques, the most extensive investigation was conducted by Baère et al., with 566 patients and 1037 tumors [39]. OS was comparable to lung metastasectomy for the first five years of treatment, mostly due to the reduced morbidity and mortality of the procedure, with patients able to tolerate serial ablations if necessary. However, Wang et al.’s prospective study reported a reduced three-year OS of 14% (compared to ~60%), which may be due to comorbidities of the study participants [40,41]. 

Prior research into the ablation of lung metastases includes tumors originating from diverse primary tumor types, with colorectal tumors being the most prevalent. Among the ablative methods, RFA has the most studies, followed by MWA [41]. Among these studies, ablative therapies demonstrated favorable local control rates, typically ranging from 80% to 90%. Representative investigations showcased promising overall survival rates, often exceeding 50% for mixed and colorectal tumors at five years post-ablation [41]. 

When compared to other techniques, such as MWA and cryoablation, RFA has shown comparable efficacy and safety and, therefore, no specific technique can be recommended for tumor ablation. Secondary factors, such as targetability with equipment, available imaging modalities, and operator expertise, are major decision factors that can impact outcomes. Alternative ablation techniques require more data regarding safety and efficacy with long-term outcomes.

## 5. Microwave Ablation

### 5.1. Mechanism and Technique

MWA is an interventional therapy used to treat solid tumors. Similar to RFA, the technique relies on thermoablation of malignant tissues. The methodology of MWA differs in that the heat is not generated by an electric current, but rather through the resonance frequency of water to absorb microwaves to convert electromagnetic energy into thermal energy [10,42]. This provides the added benefit that surgical clips or pacemakers are not contraindications. MWA is considered a safe and effective treatment option for certain types and sizes of tumors, particularly for patients who may not be candidates for surgery or who prefer a less invasive approach. It can provide localized treatment with minimal damage to surrounding healthy tissue and typically has a shorter recovery time compared to traditional surgery [43]. 

Prior to ablation, the tumor needs localization with image guidance, such as ultrasound, CT scans, or MRI, for probe placement and microwave targeting. Once the tumor is located, a small probe (also called an antenna or applicator) is inserted directly into the tumor tissue through a small incision in the skin. The probe is positioned within the tumor under imaging guidance. A coolant, either CO_2_ or fluid, is passed through the needle to prevent skin burns. Each cycle of microwaves on tissue can produce a discrete focus of approximately 1.6 cm of necrosis for 120 s at 60 W (image-guided ablation). Temperatures achieved during the procedure cause coagulative necrosis, effectively killing the tumor cells. The extent of the ablation depends on factors such as the duration and intensity of the microwave energy, as well as the size and location of the tumor [44].

### 5.2. Hepatic Ablation

To be considered for renal ablation therapy with curative intent, there must be a single nodule < 5 cm in diameter or up to three nodules < 3 cm each, and no portal vein cancerous thrombus or metastasis. For palliative MWA treatment, patients can have hepatic lesions > 5 cm or multiple lesions (including BCLC B stage HCC), a small extrahepatic tumor burden, and be able to tolerate the MWA procedure [45].

Barcelona Clinic Liver Cancer guidelines recommend solitary HCC ≤ 2 cm as standard therapy for non-transplant candidates without vascular or extrahepatic dissemination [46]. As discussed above, RFA and MWA have been used interchangeably among institutions and providers for percutaneous hepatic ablation [9,47]. MWA is often preferred due to the aforementioned advantages, including multiple simultaneous ablations, larger ablation zones with higher temperatures, no electrical impedance, and a reduced heat sink effect. Despite the theoretical differences, randomized trials have demonstrated comparable HCC treatment outcomes using RFA and MWA [48,49].

Recent advances in RFA and MWA therapy for HCC include combination therapy. MWA (or RFA) can be performed in conjunction with embolization therapies, such as transarterial chemo- or radio-embolization (TACE and TARE). The survival benefit is most significant for larger lesions > 3 cm. The hypothesis of the added benefit to survival is that the additional stresses from radiation, thermal, and ischemic metabolic derangements prevent the escape pathways from apoptosis and force cells into necrosis and necroptosis [21]. 

The additional benefit is believed to be due to the enhanced immunologic response from the surrounding parenchymal tissue. Both MWA and TACE/TARE have been independently shown to increase CD4^+^ and CD8^+^ T cell activation with cytokine release in response to tissue damage. Combined immunotherapies are designed to potentiate this response to improve cellular tumor immunity to improve survival. While there is conflicting data on whether these combination therapies improve OS, there are still ongoing trials with neoadjuvant therapies, such as sorafenib, pembrolizumab, nivolumab, atezolizumab, and bevacizumab [22].

### 5.3. Renal Ablation

MWA is a well-studied technique for the treatment of renal cell carcinoma (RCC). Zhou et al.’s retrospective study of T1N0M0 RCC MWA has comparable treatment responses to RFA or cryoablation, but with the advantage of shorter procedure times and less associated sedation [50]. Additionally, several other studies have indicated similar therapeutic efficacy and preservation of renal function across MWA, RFA, and cryoablation modalities [51,52]. However, these procedures can carry a relative high rate of complications. In a retrospective analysis involving 111 patients with renal tumors who underwent 105 US-guided percutaneous MWA procedures, a complication rate of 24.8% was observed [53]. Among these studies, the major complications included hydrothorax and bowel injury. Minor complications included microscopic hematuria, mild thermal injury to the psoas muscle, perirenal hematoma, diarrhea, abdominal distension, lower-extremity edema, and pelvicalyceal thermal injury.

### 5.4. Lung Ablation

Recent comparative studies have demonstrated that outcomes and safety profiles between RFA and MWA are similar [54,55,56,57,58,59,60]. However, Yuan et al. conducted a meta-analysis comparing RFA and MWA, pooling 3432 patients [61]. RFA did have an associated higher OS when compared to MWA for primary tumors, as well as better survival for patients with pulmonary metastasis. MWA is similarly well tolerated and carries a mortality rate of less than 1%. Minor complications include pneumothorax, pleural effusion, phrenic nerve injury, tumor implantation, and intraparenchymal hemorrhage. Major complications include bronchopleural fistula, pulmonary artery pseudoaneurysm, air embolism, and diffuse pneumonitis. The complication rate for the MWA procedure can be as high as 20.6% [45,46,47].

### 5.5. Pancreatic Ablation

Similar to RFA, MWA is a palliative procedure to reduce tumor burden in advanced neoplasms and can function as an adjunct to chemotherapy and radiation [62]. Ablation to the pancreas is high risk, and safety and efficacy trials have shown similar complications to RFA [62]. Complications include pancreatitis, pancreatic duct fistulas/leaks, and pseudocysts. While OS can be as high as 80% one-year post-MWA, there is a low complete tumor response rate, as measured by RECIST 1.1 or Choi criteria, as well as local tumor progression measured at nine months [63]. For MWA, despite imperfect tumor ablation and cytoreduction, quality of life (QOL) analysis shows significant improvements when compared to pre-procedural measurements [64]. Further studies of the comparative advantage of MWA with other palliative therapies, including chemotherapy or celiac plexus pain relief, are needed for more thorough recommendations of the optimal palliative approach. According to recent evidence, patients will likely benefit from a combinatorial approach to palliative care.

### 5.6. Adrenal Ablation

The American Association of Endocrine Surgeons advocates for adrenalectomy for malignant tumors, citing limited evidence of RFA or MWA to make stronger recommendations to its comparative efficacy. However, there is a growing literature showing localized ablation of primary and metastatic adrenal lesions in patients who are not surgical candidates [65,66,67]. RFA and MWA have ablated aldosterone-producing and cortisol-secreting adenomas with cure rates as high as 75–100% [66]. Ablation is also favorable to unilateral adrenalectomy for the treatment of aldosterone-producing adenomas associated with hypertension [68,69,70]. In the palliative therapy of metastatic adrenal tumors, ablation can be achieved with RFA or MWA, with residual tumors visualized in <25% of patients [66]. With the evidence of successful tumor ablation, the technique has been adapted to treat non-oncological conditions, including Cushing’s syndrome. Larger cohort and randomized control trials are required before MWA ablation can be recommended as a standard of care for patients that are candidates for surgical resection.

### 5.7. Parathyroid Ablation

MWA can be used to target nodules in both the parathyroid glands. The first-line therapy for parathyroid malignancies is surgical resection, with a low rate of recurrence [71]. Parathyroid lesions can be difficult targets for ablation, as these microadenomas are often undetectable with standard imaging techniques. For parathyroid nodules, both malignant and non-malignant masses are treated similarly, as the final pathology from the resected sample is used for final classification. Most of the literature discussing parathyroid ablation addresses primary hyperparathyroidism (pHPT), indicating the success of the technique, which would be used in the setting of malignant parathyroid adenocarcinomas.

A large-scale multicenter study enrolled 119 patients with a total of 134 parathyroid nodules, with an observed cure rate of 89.9%. Notably, the ablation zone had disappeared in the majority of patients by the 12-month follow-up [72]. Compared to the reported 95% cure rate associated with surgical resections, thermal ablation displayed a slightly lower cure rate, primarily attributed to factors such as false puncture or incomplete ablation. Similar to surgical resection, intraoperative intact parathyroid hormone monitoring could be employed as a supplementary measure to ensure complete ablation.

Currently, there are no definitive criteria for selecting between RFA and MWA for pHPT treatment, with operator preference playing a significant role. Other thermal ablation techniques, such as high-intensity focused ultrasound and laser ablation, have also been explored for pHPT treatment, but their efficacy requires further validation [73,74]. In a larger multicenter study of 229 MWA and 453 resected patients, MWA had comparable OS and progression-free survival at 1, 3, and 5 years, but with lower rates of complications, shorter procedural times, and shorter durations of hospital stays [75].

Transient hoarseness is a common complication following thermal ablation of pHPT. However, the rate of permanent recurrent laryngeal nerve-injury-induced hoarseness remains lower compared to thyroidectomy or parathyroidectomy procedures. This could be attributed to the efficient hydro-dissection technology employed to reduce thermal stimulation to the recurrent laryngeal nerve, coupled with accurate puncture and ablation monitored by ultrasound [75,76]. Overall, patients are more satisfied with an ablation procedure, when possible, in part due to ablation being a faster procedure, with less scarring, reduced hospitalization, and a lower cost [77].

### 5.8. Thyroid Ablation

Papillary thyroid carcinoma is the most common thyroid malignancy, comprising 70% of malignant thyroid nodules. Microcarcinomas are defined as nodules that are less than 1 cm in size, and they carry a favorable prognosis and very low risk of disease-specific mortality. For these nodules, treatment consists of thyroidectomy or surveillance. The population who are at high surgical risk or elect for nonoperative management may be candidates for thyroid ablation with MWA (or RFA). A meta-analysis of thyroid ablation showed that MWA (and RFA) were effective at significantly reducing the volume of the nodule [73]. Approximately 56.5% of patients experienced a complete disappearance of the nodule. Patients experienced an average nodule volume reduction of 95.3%. Of note, RFA and MWA performed similarly, with no recommended methodology for ablation.

The major complication rate of voice changes or hypothyroidism for ablation was low, around 0.7%. The minor complications included hoarseness, hematoma, and pain, which occurred in 2.5% of patients. With promising results, further research is exploring the possibility of ablation in other thyroid cancers, such as follicular, papillary, medullary, and anaplastic. However, there are limited data for its use in these tumors. The concern for ablation is that only half of patients had complete ablation, which increases the risk of recurrence. Therefore, this would be reserved for patients that have recurrence or are unable to undergo thyroidectomy.

### 5.9. Bone Ablation

A meta-analysis conducted in 2019, encompassing RFA, MWA, cryoablation, and magnetic resonance-guided focused ultrasound (MRgFUS), revealed that all these techniques provided pain relief in up to 91% and 95% of patients after one and three months, respectively [78]. However, MRgFUS was associated with a notable complication rate compared to the others, which demonstrated relatively higher safety profiles. Although additional comparative studies are warranted, various systematic reviews, clinical trials, and cohort studies have underscored the safety and efficacy of RFA, MWA, and cryoablation in managing primary and metastatic bone tumors [79].

MWA stands out due to its quicker coagulation times, enhanced depth of penetration, and decreased osseous impedance, in comparison to alternative ablative methods. These features render it particularly beneficial for addressing deep and sizable lesions, with mitigated heat sink effects and a reduced risk of charring buildup. Nonetheless, it is worth noting that current manufacturer guidelines primarily focus on soft-tissue tumors, necessitating further optimization of parameters specifically tailored for bone tumor treatments [80].

## 6. Cryoablation

### 6.1. Mechanism and Technique

Cryoablation is in the category of thermal ablation, except it uses freezing for both chemical and biomechanical stress to the tissue to induce cell death. The probe is inserted proximal to the malignant tissue and rapidly cooled using the Joule–Thompson effect of expanding gas within the probe. When high-pressure gas (typically argon) at room temperature reaches the distal end of the cryoprobe, it is forced through a narrow opening (throttle) and rapidly expands to atmospheric pressure. This rapid expansion cools the gas (Joule–Thompson effect), and the cooling effect is quickly transferred to the metallic walls of the cryoprobe via convection and conduction. The depressurized gas is then vented out through the hub of the needle. To warm the cryoprobe and thaw the tissue, high-pressure helium is used, which heats the cryoprobe as it expands to atmospheric pressure [81].

The tissue is damaged by ice crystal formation within the cell, creating membrane pores and organelle destruction. Even without crystal formation, the area surrounding the frozen tissue will undergo apoptosis via a combination of osmotic stress and caspase activation, which results in cell shrinkage, membrane blebbing, chromatin condensation, and genomic fragmentation. Concomitant indirect cell injury occurs through vascular compromise to the surrounding tissue. Hypothermic tissue results in platelet dysfunction, endothelial damage, and microvascular thrombus formation, which provide secondary ischemic damage to the cells [82].

Cryoablation-based combination therapies have been extensively tested in preclinical and clinical trials to improve current ablation regimens. Recent studies have shown that cryoablation can trigger a systemic anti-tumor immune response, opening new avenues for immuno-cryotherapy. The development of multifunctional nanocarriers is pivotal for expanding cryoablation in cancer treatment, as these nanomaterials enhance cryo-cell death and customize ice formation to fit irregular tumor shapes. Image-guided nanoparticle seeding can optimize ice formation within tumors, while protecting healthy tissues. This nanoparticle-based combinational cryotherapy can address challenges, such as drug resistance, systemic toxicity, and uneven cancer cell destruction, making it superior to traditional chemotherapy or cryoablation alone [83].

### 6.2. Hepatic Ablation

Cryoablation offers a potentially curative option for patients with small tumors. Cha et al. treated nonsurgical patients with HCC and found cryoablation to be effective in HCC and metastatic colorectal adenocarcinoma [84]. Compared to patients that did receive hepatic resections, there was no difference in OS, but with fewer complications. The effectiveness of cryoablation diminishes for liver tumors larger than 4 cm, which have higher rates of local progression and complications [2]. In a study involving 98 patients treated with hepatic cryoablation, 11% had major complications, with significantly higher rates in larger treated areas (>30 cm^2^) [85]. Caution should be used when choosing cryoablation in hepatic tumors. Hepatic cryoablation in animal models has shown systemic complications. Adverse effects of hepatic cryoablation vary in severity and frequency. Liquid nitrogen ablation has a high adverse effect rate (up to 40%) and includes complications such as liver parenchyma fracture, cryoshock, myocardial infarction, biliary fistula, hemorrhage, thrombocytopenia, and coagulopathy [86,87]. The mortality rate for hepatic cryoablation is approximately 1.5%, comparable to liver resection. Percutaneous cryoablation has fewer and milder adverse effects, but serious complications, such as hepatic failure, rupture, and cold shock, can still occur [88].

Approximately 6% of patients experienced serious complications, including cryoshock, hepatic hemorrhage, liver abscess, gastric bleeding, intestinal fistulas, and acute hepatic failure resulting in death. Cryoshock was associated with large areas of ice formation or extended freezing times, involving renal failure, disseminated intravascular coagulation, and adult respiratory distress syndrome. Another serious complication that should be considered is an argon–helium gas embolism [89]. Lastly, tumor seeding is detected in 0.8% of patients within six months post-cryoablation [90].

Despite these risks, cryoablation is safe and effective for smaller hepatic tumors near other organs, such as the gallbladder, achieving high technical success and favorable clinical outcomes [91]. The operator needs to consider the relevant risk factors for the patient in selecting the optimal ablation technique, as cryoablation can be a useful tool in hepatic tumor ablation.

### 6.3. Renal Ablation

Cryoablation emerges as a promising treatment avenue for kidney cancer, particularly for managing small masses (<3 cm), as indicated by recent literature on thermal ablation therapies. However, a meta-analysis by the Agency for Healthcare Research and Quality (AHRQ), comparing partial nephrectomy and thermal ablation therapies, revealed comparable cancer-specific survival rates without clear superiority for either modality. While partial nephrectomy exhibited better overall survival, this finding may be influenced by selection biases favoring surgical candidates and individual patient factors impacting mortality risks [92]. Tailoring treatment strategies based on individual risk factors remains crucial. Current guidelines from the American Urological Association (AUA) suggest thermal ablation as a moderate recommendation for suitable patients, supported by grade C evidence.

In comparing thermal ablation methods, such as cryoablation, RFA, and MWA, no significant discrepancies in treatment outcomes, including complications, metastatic progression, or cancer-specific survival, have been noted [51,52]. However, a multicenter study contrasting RFA and cryoablation revealed higher primary efficacy rates and better five-year survival with cryoablation for tumors sized between 4 cm and 7 cm [93]. Cryoablation’s advantage lies in its continuous monitoring capability during the procedure, ensuring adequate tumor coverage and margins, which may require fewer ablations compared to RFA [83]. Additionally, cryoablation offers better control over the shape of the ablation zone, overcoming limitations associated with charring in RFA due to its biophysics. Furthermore, analyses involving thousands of patients suggest that cryoablation holds lower local recurrence rates compared to RFA.

While thermal ablation demonstrates comparable oncological efficacy to nephrectomy for cT1a kidney tumors while preserving renal function, its effectiveness diminishes for larger tumors (2–4 cm) [94]. Studies involving thousands of patients underscore that RFA and cryoablation may not be as effective as partial nephrectomy in ensuring overall survival for tumors in this size range [94]. Cryoablation, particularly for tumors 2 cm or smaller, exhibits comparable overall survival to partial nephrectomy, highlighting its potential as an alternative treatment option. Despite similar oncological outcomes, cryoablation appears to offer better overall prognosis compared to RFA when juxtaposed with the current standard of care of partial nephrectomy.

### 6.4. Lung Ablation

Compared to thermal ablation, cryoablation offers several advantages: the ability to achieve larger tumor ablation volumes by using multiple cryoablation needles, enhanced visibility of the ablation area under CT scan for more accurate and effective treatment, and improved patient tolerance [95]. Cryoablation is safer with tumors positioned near blood vessels or bronchi, as acellular collagen structures in frozen tissue are preserved. A common complication of cryoablation is puncture bleeding, with modern equipment containing a heated probe shaft to mitigate this issue.

Cryoablation has been studied for treating both early and advanced non-small-cell lung cancer (NSCLC). Moore et al. conducted a retrospective study on T1N0M0 NSCLC patients treated with cryoablation, reporting a five-year survival rate of 67.8% and a five-year cancer-specific survival rate of 56.6%, with a combined local and regional recurrence rate of 36.2% [96]. Lyons et al. evaluated cryoablation for advanced NSCLC, finding the marginal probability of local recurrence at 1, 2, and 3 years after ablation to be 11.4%, 11.4%, and 38.1%, respectively, with 19 patients developing pneumothorax and 7 requiring thoracic drainage [97]. Pusceddu et al. reported successful completion of cryoablation treatments for primary and secondary lung tumors, with a technical success rate of 92%, and Gao et al. observed a main technical effective rate of 100% after a one-month follow-up for patients with advanced NSCLC after failure of radiotherapy and chemotherapy [98,99]. Yang et al. conducted a multicenter, randomized controlled trial, demonstrating a high ice hockey coverage rate (ICR) of 98.66% and a disease control rate (DCR) of 95% for cryoablation in stage III–IV NSCLC patients [100].

A significant advancement in interventional oncology is the combination of local ablation and systemic therapy. Thermal stress in cryoablation potentiates antigen presentation, likely through protein misfolding and ice-mediated fragmentation, and increases the anti-tumor immunity. This process is not well understood, and further research may implicate the role of cryoablation in mediating immunotherapy of lung cancer [95].

### 6.5. Breast Ablation

Cryoablation has seen increasing utilization in the treatment of breast cancer across various stages, ranging from early to advanced disease. The pursuit of favorable cosmetic outcomes has prompted a shift toward breast-conserving, minimally invasive approaches, such as radiotherapy and ablation therapies, instead of traditional surgery. Numerous case studies have underscored the safety and efficacy of cryoablation for treating solitary, small-sized tumors [101,102]. However, for cases involving multifocal lobular carcinoma, cryoablation is generally discouraged due to the elevated risk of fat necrosis and damage to healthy tissues caused by prolonged freezing periods [103]. Clinical trials, including a phase II study focusing on early-stage breast cancer tumors smaller than 2 cm, have reported high overall success rates, particularly in patients without multifocal cancer [104]. Similarly, recent prospective trials involving patients with breast tumors less than 1.5 cm have indicated low recurrence rates and high patient satisfaction with cosmetic outcomes [102]. Careful attention should be paid to safeguard sensitive areas, such as the nipple, from potential burns during the procedure, and techniques such as saline hydro-dissection may be employed to shield the skin from the effects of the ice ball.

### 6.6. Desmoid Ablation

Cryoablation (CA) has become increasingly favored for managing both benign and malignant musculoskeletal tumors. Despite being non-malignant, extra-abdominal desmoid (EAD) tumors pose significant treatment challenges due to their propensity for high rates of local recurrence. Recent evidence suggests that cryoablation for these tumors is effective and safe [105]. Auloge et al. observed a notably high complete response rate (68.4%) in patients undergoing curative-intent treatment, surpassing rates reported in other studies, although the median follow-up period was relatively short at 18.5 months, lacking long-term data [106,107,108,109,110,111,112]. The studies demonstrating complete responses typically did not document subsequent local recurrences [106]. The potential curative role of cryoablation, particularly when achieving complete ablation, holds promise, but further long-term data are warranted to comprehensively assess its efficacy. Cryoablation offers the advantage of real-time monitoring of the ice ball using imaging, resulting in minimal complication rates. However, not all extra-abdominal sites are suitable for cryoablation, with craniofacial locations posing heightened risks of neurovascular and cutaneous complications. Common complications include injury to the common peroneal nerve and skin necrosis, particularly in the popliteal fossa [111]. Factors such as proximity of the tumor to the skin, large tumor volumes, and pre-cryoablation embolization are recognized as risk factors for skin necrosis. Mitigation strategies employed in this series include carbo- or hydro-dissection and neuromonitoring.

### 6.7. Prostate Ablation

The first clinical success cases of prostate cryoablation were conducted using whole-gland cryoablation. Modern techniques utilizing fusing MRI/US imaging have enabled a shift toward hemi-gland and focal approaches. When selecting patients for ablation, urological guidelines only recommend using low- to intermediate-risk prostate cancers because there are no head-to-head trials demonstrating comparable efficacy to surgical resection. For high-risk tumors, cryoablation should be reserved for those refusing or unable to undergo standard therapy [113]. Several small clinical studies, including from 10 to 40 patients, have shown success with MRI-guided cryoablation, with comparable oncologic outcomes to resection [114,115,116,117]. An important factor in reducing occurrences is monitoring the formation of the ice ball to ensure that there are sufficient margins.

An important technical factor in successful cryoablation is ensuring preservation of critical structures that can be damaged by thermal stress. The major complication reported is a rectourethral fistula, which is most common with whole-gland ablation. However, the most common minor complications include infection, dysuria, scrotal pain, urinary retention, and incontinence [118]. The primary strategies for reducing these complications include the implementation of hybrid MR imaging to improve the placement of the probes, real-time monitoring with temperature probes, and US visualization. To protect the urethra, a catheter is inserted and flushed with continuous saline for urethral warming. To prevent rectal damage, innovative approaches include proactive cryoprobe rectal warming, saline displacement of the rectal wall, or injecting a hydrogel into Denovilliers’ space [119,120,121]. Collectively, these adjustments have reduced the complication rate while maintaining the high levels of technical success for focal and whole-gland ablation.

## 7. Irreversible Electroporation

Electroporation, otherwise known as electro-permeabilization, is a technique commonly used in cellular biology to induce transient membrane pores via an electrical field, which could be used to introduce large molecules, such as biologic drugs or DNA. Irreversible electroporation (IRE) is the technique translated into interventional oncology to ablate tumors. The voltage and frequency of the pulses are intense enough to stress the local cells into forming large membrane defects that result in apoptosis [10,122]. IRE also induces an immune response, which can enhance the body’s ability to recognize and eliminate cancer cells. This immune response may contribute to the long-term effectiveness of the treatment by targeting residual tumor cells and preventing recurrence. This method is distinct from other thermal ablation techniques, such as MWA or RFA, in that the effectiveness of the ablation is independent of the thermal energy produced. IRE is particularly advantageous for treating tumors near sensitive structures, such as blood vessels, nerves, or ducts, where thermal-based ablation techniques may pose a higher risk of damage. IRE can be scaled down to a small size, and with proper localization, IRE is also considered a promising option for tumors that are not suitable for surgical resection or thermal ablation due to their location or size [123].

Variations of this technique have been developed to improve the efficiency of pulse delivery. Because of the voltage applied, the patient requires paralytics to prevent muscle contracture, and cardiac synchronization to prevent cardiac arrythmias. Ultra-short, high-frequency bipolar pulses, known as H-FIRE, obviate the need for cardiac synchronization and paralytics, while maintaining a measurable effect on cell death. 

IRE has unique characteristics that improve the immunogenic potential. Unlike thermal ablation methods, the vasculature is largely preserved. The intact blood supply and microvasculature allows for the continued infiltration of antigen-presenting cells (APCs) and facilitates the migration of tumor antigens in the lymphatic system. The high-voltage current causes pores sufficient for intracellular release of various components that serve as damage-associated molecular patterns for macrophage activation. This creates an M1 macrophage phenotype consistent with generating an anti-tumor environment [124]. This generates a sustained T-cell immune memory that reduces tumor growth beyond the primary site of ablation [125]. Additionally, the pulses induce apoptosis in immune-suppressing regulatory T-cells and myeloid-derived suppressor cells [126]. Lastly, IRE promotes increased porousness of connective tissue, which further facilitates immune migration. Collectively, there is evidence of a systemic advantage to IRE in improving the systemic immune response in the setting of metastatic cancer. 

### 7.1. Hepatic Ablation

Liver lesions located centrally may not be suitable candidates for surgical resection or thermal ablation due to their proximity to crucial structures, such as the main bile ducts and portal vein. Irreversible electroporation (IRE) presents a promising alternative by preserving these vital structures, making it a particularly viable option for such cases. While there are no strict size limitations, IRE appears most effective for tumors measuring 3 cm or less in diameter. Beyond this threshold, multiple overlapping ablations may be necessary, leading to reduced efficacy. Numerous studies have investigated the application of IRE for both primary and secondary liver malignancies. Scheffer et al. were pioneers in demonstrating the feasibility of IRE for the radical ablation of human tumors. Their study, known as the COLDFIRE-1 trial, showcased the ability of IRE to induce complete macroscopic tumor nonviability in colorectal liver metastases, as validated by 5-triphenyl tetrazolium chloride vitality stain.

The efficacy outcomes of hepatic irreversible electroporation (IRE) display significant variability in the current literature, spanning from 45.5% to 100%. This wide range can be attributed to the diversity of treated tumors and patient demographics. Additionally, physicians performing IRE exhibit a learning curve, with complication and recurrence rates stabilizing after approximately 100 cases. This suggests that reported efficacy rates may improve with accumulating experience. The ongoing COLDFIRE-2 trial, a phase I/II investigation of safety and efficacy for centrally located colorectal liver metastases (CRLM; NCT02082782), holds promise for further insights [127]. Notably, preliminary results from this trial demonstrate IRE’s effectiveness in managing even large liver tumors.

Resection remains the sole potentially curative option for perihilar cholangiocarcinoma (PHC), yet only a small proportion of patients are candidates for resection upon diagnosis [56]. Unresectable PHC, comprising locally advanced and metastatic forms, carries a bleak prognosis, with a median overall survival of three to six months. Irreversible electroporation (IRE) demonstrates safety and feasibility near main bile ducts and major vascular structures, making it a potential treatment avenue for locally advanced PHC, characterized by specific imaging criteria. While literature on IRE in PHC is scarce, existing data on its safety for other perihilar liver malignancies are promising, supported by case reports and small series demonstrating successful outcomes and minimal morbidity.

### 7.2. Pancreatic Ablation

IRE serves a distinct role in pancreatic cancer treatment, offering primary tumor control and margin accentuation in combination with resection for borderline resectable tumors. The choice between an open or percutaneous approach and CT or US guidance depends on the physician’s preference and experience, as direct comparisons are lacking. Safety concerns include gastrointestinal complications, such as nausea, vomiting, and diarrhea, along with duodenal ulcers and perforations, particularly if the intestine is near the ablation zone. Elevated amylase and lipase levels, pancreatitis, and vessel-related complications, such as vessel narrowing or portal vein thrombosis, may occur [128]. Post-IRE, mild-to-moderate blood pressure increases are common, especially during pancreatic procedures, possibly due to stimulation of the autonomous nervous system. While performing IRE near metallic objects is generally discouraged, some centers have safely conducted procedures with metal stents within the ablation zone.

In contrast to hepatic IRE, mortality associated with pancreatic IRE has been documented [128]. Kluger et al. observed six deaths within ninety days post-IRE, with three deemed directly IRE-related: one due to upper gastrointestinal bleeding and portal vein thrombosis, another from intraperitoneal hemorrhage necessitating gastroduodenal artery embolization leading to multisystem organ failure, and one from duodenal and bile duct necrosis [129]. Martin et al. also reported 3 deaths within 90 days after IRE, including upper gastrointestinal bleeding from an IRE-induced duodenal ulcer, liver failure in a patient with pre-existing complete portal vein thrombosis, and pulmonary embolism [130].

Regarding efficacy, data from various case series inform current understanding. Martin et al.’s extensive study involving 200 LAPC patients treated with IRE, either for primary local control or margin accentuation combined with resection, reported a median OS of 24.9 months from diagnosis. Narayanan et al. reported a median OS of 27.0 months from diagnosis and 14.2 months from IRE for primary local control in 50 patients, all of whom received chemotherapy prior to IRE, with 3 patients undergoing surgical resection post-IRE, resulting in negative margins and tumor necrosis. In the PANFIRE study, 25 LAPC patients were treated with IRE for primary local control, yielding a median OS of 17.0 months from diagnosis and 11.0 months from IRE [131]. The shorter survival in the PANFIRE study, compared to others, may stem from fewer patients receiving chemotherapy pre-IRE, which typically improves survival and selects patients suitable for IRE by filtering out those with rapid disease progression.

### 7.3. Renal Ablation

The ability to target renal cancers in traditional thermal ablation methods is dependent on anatomic location. Tumors located near the central hilum or vascular pedicles have a higher risk of structural damage. IRE can reduce these risks because of the non-thermal mechanism, which preserves acellular tissue. Despite this advantage, higher-intensity settings can still lead to ureter strictures and transmural necrosis [132]. IRE lacks significant clinical testing, with Pech et al. completing IRE on RCC in six patients [133]. Canvasser et al. conducted the largest study of 42 patients, with a technical success rate of 93% with no major complications [134]. Cohort and randomized prospective studies are still needed to confirm the oncological efficacy of IRE in treating renal cell carcinoma.

### 7.4. Prostate Ablation

An initial concepted application of IRA was for prostate cancer, with pre-clinical models showing the ability to preserve neurovascular bundles, the urethra, and the rectum [135,136]. Neal et al. conducted the first human prostate IRE in two patients, with subsequent prostatectomy showing no malignant tissue within the ablation zone [137]. Studies by Blazevski et al. and Wang et al. included 50 and 117 patients with d’Amico criteria low/intermediate-risk prostate cancers, respectively, demonstrating a safe technique with no significant adverse events observed [138,139,140]. Recurrence or residual tumor rates were minimal at 6 and 12 months post-IRE. Disease-free survival at three years was impressive at 96.75%. Considering the conceptual nature of IRE as partial prostate ablation, it is most appropriate for patients necessitating focal therapy. However, approximately 10–15% of IRE-treated patients develop new or additional lesions outside the ablation zone during follow-up. Fusion of 68Ga-prostate-specific membrane antigen positron emission tomography and multiparametric magnetic resonance imaging could enhance detection of potential multifocal tumors, while advancements in genetic and epigenetic markers show promise [141,142].

## 8. High-Intensity Focused Ultrasound

### 8.1. Mechanism and Technique

The HIFU beam can traverse through the skin and surrounding tissues safely, concentrating its energy on a specific area, typically up to 3–4 cm in diameter for tumor treatment. This focused ultrasound beam penetrates through the skin and tissues to induce necrosis in a localized region, often situated deep within the body. The region directly targeted by the beam undergoes coagulative necrosis, effectively ablating the tumor. This process creates a distinct demarcation between the necrotic area and the adjacent healthy tissue, typically within a boundary width of no more than 50 µm [143].

The fundamental mechanisms underlying tissue damage caused by HIFU involve tissue coagulative thermal necrosis, which arises from the absorption of ultrasound energy during tissue transmission (thermal effect) and ultrasound-induced cavitation damage [144]. The heat generated by HIFU can elevate the temperature in exposed tissue rapidly, exceeding 60 °C, leading to immediate and irreversible cell death in most cases when exposure lasts longer than 1 s [144]. The highly focused ultrasound beam concentrates its intensity at the focal point within a small volume, approximately 1 mm in diameter and 10 mm in length, thereby reducing the risk of damage to surrounding tissues [144]. Thermal tissue damage resulting from high-temperature exposure is directly proportional to the duration of exposure and exponentially related to the temperature increase [145]. Mechanical stress produced by microstreaming jets in free-flowing mediums causes cell wall pitting and can induce apoptosis [146].

The approach to HIFU ablation depends on the accessibility of the targeted organ to ultrasound [144]. For organs, such as the kidney, where access is straightforward, external transducers are used through the skin, while for areas such as the prostate, transrectal transducers are inserted into the body. For tumors in locations such as the biliary duct or esophagus, interstitial probes are utilized, inserted through the mouth and placed near the tumor [147]. Each method has specific considerations: external devices distribute energy over a large area to avoid skin burns, transrectal and interstitial devices operate at higher frequencies and lower power for closer targeting, and catheter-based ultrasound devices offer precise energy localization but are more invasive. Additionally, HIFU systems adjust for tissue inhomogeneity and can target larger volumes through mechanical or electronic transducer movement. Overall, HIFU provides a versatile and precise means of tissue ablation for various medical conditions.

### 8.2. Hepatic Ablation

HIFU in HCC has been studied in small patient populations, with promising patient outcomes. Wu et al. investigated the pathophysiologic changes after HUFU. The tumors were ablated with sharp margins, and pathology demonstrated HIFU, observing coagulative necrosis of malignant cells in the ablation zone [148]. Wu et al. studied 1038 patients after HIFU, with technical success in patients measured by decreased blood supply and no radioisotope uptake in the ablated tumor [149]. Li et al. performed HIFU on 100 liver cancer patients (62 HCC, 38 metastatic tumors), with symptomatic improvement in 87% of patients after HIFU, without any complications [150]. The benefit of HIFU is that these procedures can be achieved without anesthesia or sedation. HIFU is also used in conjunction with other palliative therapies, including transcatheter arterial chemoembolization (TACE), with synergistic benefits of increased OS relative to the independent procedures [151]. The current studies are small, but HIFU shows continued progress in effective curative and palliative treatment of HCC and solid metastatic tumors.

### 8.3. Breast Ablation

HIFU is used as a nonsurgical treatment for breast cancer in patient populations that are poor surgical candidates and prioritize tissue conservation. Compared to lumpectomy, HIFU shows decreased anesthesia needs, improved recovery, lower infection risks, and no scar formation. HIFU can target multiple malignant breast tumors, including invasive lobular carcinoma, ductal carcinoma, and mucinous adenocarcinoma, with reported coagulation necrosis rates of 88–100% [152,153,154,155,156]. These procedures have no major complications, with minor complications including local mammary edema and minimal skin burns [157]. The primary drawback is patients will require pathological samples to assess treatment margins [144]. Wu et al. performed a small, randomized, prospective trial of HIFU in patients that received chemotherapy, radiation, and tamoxifen [154]. With this treatment plan, patients experienced a five-year DFS and recurrence-free survival of 95% and 89%, respectively, with cosmetic approval in 94% of patients. HIFU will continue to be a focus of continued research in noninvasive tumor ablation.

### 8.4. Prostate Ablation

In the treatment of prostate cancer, the advantage of HIFU is the ability of the probe to target the prostate transrectally. Preliminary clinical studies following up to five years after HIFU have shown a stable reduced PSA and 60–90% negative biopsy rate [158,159,160]. Whole-prostate HIFU can reduce tumor incidence by up to 35%, with a 90% reduction in tumor volume [161]. If the prostate cancer is more advanced, HIFU is used in conjunction with high-dose brachytherapy. Similar to IRE ablation, common complications include urinary retention, infection, incontinence, urethral stenosis, impotence, rectal fistulas, and chronic pain [162]. The risks of retention can be significantly reduced by offering a transurethral resection prior to ablation. Despite these complications, patients treated with transurethral resection of the prostate and HIFU demonstrated a significant improvement in their urinary symptoms and quality of life [163]. While the American Urological Association guidelines in 2017 did not recommend HIFU as standard therapy for low-grade localized prostate cancer, several years of clinical evidence are showing that it may be a viable alternative to surgical resection. Without more data, HIFU can continue to provide an option for morbidly obese and nonsurgical patients [164].

### 8.5. Renal Ablation

RCC is a common cancer to develop in the later decades of life, often in patients that have several comorbidities that increase surgical risks for complete or partial nephrectomy. The American Urological Association guidelines recommend patient consideration for localized ablation therapies, including HIFU for masses < 3 cm, because evidence has shown decreased rates of complications and improved renal function without sacrificing the quality of tumor control [165]. Post-ablation, patients can experience up to 90% immediate pain relief, with continued tumor shrinkage up to six months after the procedure [166,167,168]. The primary limitation that would preclude HIFU would be effective percutaneous targeting. Tumors in the upper poles are subject to energy absorption by the lower ribs that may impact the degree of ablation that is possible without a laparoscopic approach [168].

### 8.6. Esophageal Ablation

HIFU was investigated for the treatment of squamous cell carcinoma (SCC) of the esophagus with clinical trials in the late 2000s. Current therapy has a low five-year OS ranging from 12% to 18%, with surgical intervention having several major complications [169]. Most of the evidence for this indication is from studies in the late 2000s, without further clinical data to support further implementation. Initial studies on 4 patients had promising results, with dysphagia improvement in 15 days [170]. The limitation of this technique is that is requires a specialized transducer and HIFU applicator that can apply high-intensity ultrasound waves intraluminal to the esophagus [169].

### 8.7. Pancreatic Ablation

Pancreatic adenocarcinoma often presents with an advanced stage, precluding surgical intervention, leading to further research into different localized tumor ablation techniques, including HIFU in combination with other palliative therapies. Multiple studies of several hundred patients have shown the ability of HIFU to reduce tumor size and improve pain symptoms in 80% of patients [171,172]. A recent randomized prospective trial of stent placement with or without HIFU showed that ablative treatments can increase average stent patency and improve OS [173]. Complications for HIFU are similar to those of other ablation techniques and include subcutaneous fat and vertebral necrosis, pain, pancreatitis, pseudocysts, and burns. Major complications include tumor-duodenal fistulas, duodenal strictures requiring stents, and second- and third-degree skin burns [174].

### 8.8. Intracranial Ablation

Glioblastoma is a challenging cancer for ablation therapy to the cranial bones, serving as a barrier to energy targeting the tumor. HIFU treatment for brain tumors faces challenges, such as difficulty in mapping temperature variations and the risk of inducing cavitation and hemorrhage in small capillary vessels at high-intensity sonication [175,176,177]. HIFU intensity above 4400 W/cm^2^ for 1 s can cause significant vascular damage and bleeding [178]. Additionally, HIFU shows potential in immunomodulatory therapy. Similar to the results of cryotherapy in other tumors, the cavitation and mechanical disruption of endogenous proteins may promote an increased immune response by the microglia and astrocytes to slow further growth [177]. Overall, while HIFU shows promise for brain tumor ablation, further clinical trials are needed to confirm its efficacy and safety.

### 8.9. Bone Ablation

High-intensity focused ultrasound (HIFU) has been used to treat bone tumors by destroying tumor microvasculature and preventing the spread of tumor cells, alleviating pain from metastatic tumors, and treating primary bone malignancies [179]. In studies involving 10–44 patients, HIFU, either alone or combined with chemotherapy, showed promising results. Follow-up over 10 to 38 months reported up to 87% survival, complete tumor regression in up to 41.7% of patients, and significant tumor volume reduction or partial necrosis/fibrosis in 8.3–33.3% of patients. Imaging confirmed inactivation of tumor foci, with local recurrence or progression in 1–3 patients and an 18.2% complication rate. Deaths from metastasis occurred in two to five stage II and III cancer patients. For primary bone tumors, HIFU resulted in complete ablation, partial to moderate response, and some progression. Additionally, HIFU significantly reduced metastatic bone pain by 69.5–92% three months post-treatment, without delaying subsequent chemotherapy [180].

## 9. Histotripsy

Histotripsy differs from thermoablation techniques that use electromagnetic radiation or ultrasound waves to conduct thermal stress to induce cell death. The advent of histotripsy as an ablative modality induces cell death through mechanical stress. Histotripsy creates targeted cavitations with large-amplitude ultrasound pulses to mechanically disintegrate tissue. These cavitations are microbubbles formed from pressure waves of high-amplitude sound waves traveling through a fluid. The pressure waves and cavitations generate sheer stress that mechanically break cells into subcellular components.

Due to differences in mechanisms, histotripsy has some technical advantages compared to other described ablation methods. Renal, hepatic, and intracranial masses can be targeted transdermally and noninvasively. Ultrasound imaging is employed to guide and monitor histotripsy, eroding tissue from a surface inward, creating sharp boundary perforations when applied to tissue–fluid interfaces, such as blood clots or cardiac tissue. Inside bulk tissues, such as tumors, histotripsy liquefies the target tissue into an acellular homogenate, which the body absorbs over one to two months, leaving behind small scars [181].

The success is independent of heat generation and thermal injury, which is amenable to microtargeting and does not cause a zone of tissue injury. One limitation is that dense connective tissue is more resistant to histotripsy, which can make dense metastatic tissue difficult to ablate. However, this also limits the spread of mechanical damage and reduces the likelihood of collateral damage to blood vessels or biliary structures.

There are several preclinical investigations conducted on tumors located in several organ systems: brain, liver, prostate cancer, renal cancer, breast cancer, pancreatic cancer, and musculoskeletal cancer. In addition to showing the ability to target and ablate tumors effectively, histotripsy has demonstrated the ability to trigger an immune response, leading to a subsequent abscopal effect in animal tumor models. While these are all potential applications, most of the trials on humans have focused on BPH and hepatic tumors. There is currently an ongoing trial to study tumor ablation in the kidney [182].

### Hepatic Ablation

This newer technology lacks significant clinical data to support its adoption over other ablative techniques. Currently, there is a Phase I human trial for palliative therapy of hepatic metastasis in eight patients with tumors less than 2.1 cm. No complications were reported, and it had an 88% technical success rate measured by MRI, with one 5 mm tumor that was missed by targeting. The targeted lesion was shown to contract over two months. Two patients with HCC and colorectal cancer showed a decrease in tumor markers and subsequent decrease in tumor size of non-targeted tumors. Similar to HIFU and cryoablation, the mechanical tumor disruption may aid in anti-tumor immunity. The abscopal effect by histotripsy has been documented in pre-clinical models but is pending further validation of the mechanism in humans [181].

## 10. Principles of Selecting an Ablation Modality

As clinical researchers have explored the applications for each ablation technique, there are several viable options for primary and metastatic tumors in various organ systems. Reviewing the literature for the methods discussed, most have high rates of technical success with low rates of major complications. In tumors that are inconspicuous on ultrasound, it is highly recommended to implement fusion imaging and CEUS to improve visualization and targeting. Selecting the best approach can be challenging, so guiding principles can help implement the optimal technique for a patient.

The best correlation for successful ablation is operator experience. Having experience using the equipment is critical for ensuring that the mass is properly targeted, and parameters are refined for sufficient ablation margins without excessive parenchymal damage. This will prevent recurrence, major complications, and require additional procedures. Marginal benefits from switching a technique will be negated by suboptimal implementation.

For masses that are superficial, a noninvasive technique should be a top consideration. The HIFU and histotripsy techniques are limited by penetration depth; therefore, masses that can be targeted, such as soft tissue, bone, thyroid, parathyroid, and prostate, will be easier to execute. Currently, HIFU should be the preferred choice, as clinical evidence for histotripsy is still being evaluated in preclinical trials. Noninvasive targeting should also be considered for hepatic masses. In most scenarios, there is a sufficient window to target the mass for ablation without interference from rib shadow or a significant penetration depth. Otherwise, intra-abdominal targeting with ultrasound of intra-abdominal masses should be carefully planned. As the depth increases, the increased probability of insufficient ablation or off-target damage increases.

Tumors adjacent to prominent vascular supply present a significant challenge for thermal ablation strategies due to the increased contribution of heat sink affecting the margins. While some mitigation strategies have been explored, such as vessel occlusion or embolization, it would be appropriate to consider thermal-independent techniques, such as IRE. Thus, heat sink is not a factor in generating an ablation zone, with also a relative advantage of reduced connective tissue damage to vessels.

Both RFA and MWA techniques have shown efficacy, with a similar profile of tumors. The greatest selection factor is tumor size. Tumors less than 3 cm can be targeted by either technique with similar rates of success. Once the tumors are greater than 3 cm, the probability of RFA with uneven ablation increases. Therefore, MWA is the more appropriate option due to the speed at which energy can be delivered for more evenly distributed tissue destruction.

For primary or metastatic tumor targeting, an additional consideration should be made toward techniques with evidence of abscopal benefits, such as IRE, cryoablation, or histotripsy. With the goal of relieving tumor burden, several of these techniques will be effective in primary ablation. The patient benefits can be potentiated by slowing tumor growth of other systemic tumors. Caution should be taken when considering cryoablation in hepatic tumors. Especially, if several metastases are targeted, the increased ablation volume increases the risk of cryoshock. In palliative patients with multiple comorbidities, this can be a fatal complication.

Beyond practical limitations of operator expertise and institution limitations in equipment, there are several functional implications to consider when choosing the best ablation technique for a patient. While these are some guiding principles, each treatment plan will be individualized to the patient, their goals of care, and collaboration of the treatment team of the oncologist and surgeons to select the best intervention.

## 11. Conclusions

When attempting to ablate primary and metastatic tumors, there are several viable options, with comparable survival and complication rates. However, each technique comes with unique advantages. RFA is a well-established technique, with data supporting its use in small primary and metastatic tumors. Larger masses or highly vascularized tissue suffer from inconsistent margins and the heat sink effect, and thus MWA or IRE is more appropriate. When possible, the least invasive approach should be considered, including HIFU and histotripsy. HIFU is adaptable to an endoscopic approach for a mass.

In addition to the technical requirements for complete ablation, when considering a localized ablation approach, the operator should consider the patient’s comorbidities, cancer type and stage, and optimal visualization modality. The future of percutaneous and noninvasive tumor ablation revolves around research of three primary factors: type of tumor destruction, controlled energy delivery, and effective tumor targeting.

The established clinical research discussed here demonstrates that there are multiple effective ways to destroy tumor tissue. Some techniques offer additional benefits beyond ablation, such as an abscopal effect of cryoablation and histotripsy to improve tumor immunity, or combinatorial therapy that impedes molecular escape mechanisms to apoptosis and cellular repair. There is no optimal type of energy delivery, rather each approach is physically tailored to the anatomical characteristics of the mass. The next generation of ablation tools are relying less on thermal techniques, only because of their challenging parameter optimization and multifactorial variables impacting ablation margins. Mechanical or electromagnetic pulses can deliver more precise margins with a higher density of energy delivery, assuming the tissue is well visualized for accurate targeting. The last element of ablation advancement is evolving imaging modalities. Masses that are indistinct on traditional US, CT, or MRI now have additional strategies for visualization. Whether evolving contrast agents, hybrid/fusion imaging, or improving live feedback monitoring, this will improve the technical success and morbidity of these procedures, while expanding the pool of eligible patients. Tumor ablation will continue to evolve as an integral part of cancer therapy to improve patient outcomes.

## Figures and Tables

**Table 1:** Comparison of the advantages and disadvantages of imaging-guidance modalities for ablation therapy.

**Image Guidance**	**Advantages**	**Disadvantages**
Ultrasound	• Real-time feedback	• Limited tissue differentiation
	• Doppler capabilities	• Visualization depth
	• Portability	• Gas distortion
	• Low cost	
	• General availability	
Contrast-enchanced ultrasound	• Improved dynamic range	• Single-plane image
	• Improved tumor detection	• Risk of allergy and pulmonary HTN
	• Quick and cost-effective	• Needs to be able to hold breath
CT	• Quick acquisition	• Radiation exposure
	• Paired with fluoroscopy and US	• Limited real-time imaging
	• Cone beam for higher resolution	• Contrast limitations with renal insufficiency
	• 3D reconstruction available	• Limitation with isodense targets
MRI	• Best tumor/tissue distinction	• High cost
	• 3D reconstruction	• Limited availability
	• Small mass resolution	• MRI-compatible tools required
	• Thermal monitoring	• More skilled procedure
		• Artifact susceptibility
**Hybrid Imaging**	**Advantages**	**Disadvantages**
Real-time CT/MR sonography	• Instant reference to CT/MR scans	• Needs software and US-compatible systems
	• Improved localization	• Inconsistencies between US and CT/MR alignment
	• Target masses poorly visualized on US	• Increased time and cost
PET-CT	• Tumor metabolic signature	• Faulty signatures
	• Detect isodense masses	• Confounded by inflammatory conditions
	• Combination scanners increase speed and accuracy	• Low resolution relative to CT
	• Better mass classification	• Reduced signals in diabetes and hyperglycemia
	• Incidentalomas	
SPECT-CT	• Improved detection over 131-I whole-body scan	• Less sensitive than PET
	• Faster than PET	• Lower resolution than PET
	• Less radiation than PET	• Fewer tracer options than PET
	• Cost-effective and more prevalent vs. PET	

**Table 2:** Comparison of the advantages and disadvantages of the various ablation techniques.

Ablation	Advantages	Disadvantages
RFA	• Cost-effective	• Limited real-time feedback
	• Versatile to tumor type	• Pacemaker interference
	• Validated in most tumor types	• General anesthesia common
	• Can use multiple probes	• Risk of ground pad burns
		• Targeting smaller tumors for safe margins
Cryoablation	• Visible ablation margins	• Ablation consistency
	• No pacemaker interference	• Tissue thermal conduction limits efficacy
	• Phase shift measurable on imaging modalities	• Long ablation times
	• Increased anti-tumor immunity	• Risk of coagulopathy and hemorrhage
		• Risk of disseminated intravascular coagulation (DIC)
MWA	• Higher ablation temperatures vs. RFA	• Requires general anesthesia
	• Large ablation zones	• Unable to monitor tissue impedance
	• Fast and short ablation pulses	• More technically challenging
	• Capable of perivascular targeting	• Proactive cooling to prevent wire overheating
	• Cost-effective	• Requires larger probes for energy delivery
IRE	• Short ablation times	• Requires general anesthesia with paralytic agents
	• Defined ablation zones	• Risk of cardiac arrhythmias
	• Temperature independent	• Less validated than other techniques
HIFU	• Non-radiative modality	• Requires patient cooperation
	• Noninvasive modality	• Risk of thermal injuries
	• Extracorporeal	• Extended treatment duration
Histotripsy	• Minimal thermal generation	• Specific equipment requirements
	• Noninvasive option	• Limited by pneumatic interference
	• Atraumatic	• Challenging targeting for deep tumors or large body habitus
	• High-resolution targeting	• Risk of thromboembolism
	• Well-demarcated boundaries	• Limited efficacy in thick connective tissue
	• Real-time feedback	

## References

[1] McGahan J.P., van Raalte V.A., van Sonnenberg E., McMullen W.N., Solbiati L., Livraghi T., Müeller P.R., Silverman S.G. (2005). History of Ablation. Tumor Ablation: Principles and Practice.

[2] Glazer D.I., Tatli S., Shyn P.B., Vangel M.G., Tuncali K., Silverman S.G. (2017). Percutaneous Image-Guided Cryoablation of Hepatic Tumors: Single-Center Experience with Intermediate to Long-Term Outcomes. AJR Am. J. Roentgenol..

[3] Wu J., Yu J., Fowlkes J.B., Liang P., Nolsøe C.P. (2023). US-guided ablation of tumors–Where is it used and how did we get there. Med-X.

[4] Goldberg S.N., Gazelle G.S., Mueller P.R. (2000). Thermal ablation therapy for focal malignancy: A unified approach to underlying principles, techniques, and diagnostic imaging guidance. AJR Am. J. Roentgenol..

[5] Floridi C., Radaelli A., Abi-Jaoudeh N., Grass M., Lin M.D., Chiaradia M., Geschwind J.-F., Kobeiter H., Squillaci E., Maleux G. (2014). C-arm cone-beam computed tomography in interventional oncology: Technical aspects and clinical applications. Radiol. Med..

[6] Chehab M., Kouri B.E., Miller M.J., Venkatesan A.M. (2023). Image Fusion Technology in Interventional Radiology. Tech. Vasc. Interv. Radiol..

[7] Venkatesan A.M., Kadoury S., Abi-Jaoudeh N., Levy E.B., Maass-Moreno R., Krücker J., Dalal S., Xu S., Glossop N., Wood B.J. (2011). Real-time FDG PET Guidance during Biopsies and Radiofrequency Ablation Using Multimodality Fusion with Electromagnetic Navigation. Radiology.

[8] Manson E.N., Hasford F., Inkoom S., Gedel A.M. (2020). Integrating image fusion with nanoparticle contrast agents for diagnosis: A review. Egypt. J. Radiol. Nucl. Med..

[9] Mansur A., Garg T., Shrigiriwar A., Etezadi V., Georgiades C., Habibollahi P., Huber T.C., Camacho J.C., Nour S.G., Sag A.A. (2022). Image-Guided Percutaneous Ablation for Primary and Metastatic Tumors. Diagnostics.

[10] Ahmed M., Brace C.L., Lee F.T., Goldberg S.N. (2011). Principles of and Advances in Percutaneous Ablation. Radiology.

[11] Goldberg S.N., Solbiati L., Hahn P.F., Cosman E., Conrad J.E., Fogle R., Gazelle G.S. (1998). Large-volume tissue ablation with radio frequency by using a clustered, internally cooled electrode technique: Laboratory and clinical experience in liver metastases. Radiology.

[12] Pereira P.L., Trübenbach J., Schenk M., Subke J., Kroeber S., Schaefer I., Remy C.T., Schmidt D., Brieger J., Claussen C.D. (2004). Radiofrequency ablation: In vivo comparison of four commercially available devices in pig livers. Radiology.

[13] Lorentzen T. (1996). The loop electrode: In vitro evaluation of a device for ultrasound-guided interstitial tissue ablation using radiofrequency electrosurgery. Acad. Radiol..

[14] Fang Z., Zhang B., Zhang W., Fei M., Ma S., Li X., Sun X., Jia L., Su Z. (2017). Current Solutions for the Heat-Sink Effect of Blood Vessels with Radiofrequency Ablation: A Review and Future Work. Advanced Computational Methods in Life System Modeling and Simulation.

[15] Cha J., Kim Y.-S., Rhim H., Lim H.K., Choi D., Lee M.W. (2011). Radiofrequency ablation using a new type of internally cooled electrode with an adjustable active tip: An experimental study in ex vivo bovine and in vivo porcine livers. Eur. J. Radiol..

[16] Yoon J.H., Lee J.M., Hwang E.J., Hwang I.P., Baek J., Han J.K., Choi B.I. (2014). Monopolar Radiofrequency Ablation Using a Dual-Switching System and a Separable Clustered Electrode: Evaluation of the In Vivo Efficiency. Korean J. Radiol..

[17] Zhu M., Sun Z., Ng C.K. (2017). Image-guided thermal ablation with MR-based thermometry. Quant. Imaging Med. Surg..

[18] Zhang K., Ferrero A., In M.-H., Favazza C.P. (2024). Thermometry mapping during CT-guided thermal ablations: Proof of feasibility and internal validation using spectral CT. Phys. Med. Biol..

[19] Zane K.E., Makary M.S. (2021). Locoregional Therapies for Hepatocellular Carcinoma with Portal Vein Tumor Thrombosis. Cancers.

[20] Makary M.S., Ramsell S., Miller E., Beal E.W., Dowell J.D. (2021). Hepatocellular carcinoma locoregional therapies: Outcomes and future horizons. World J. Gastroenterol..

[21] Zane K.E., Nagib P.B., Jalil S., Mumtaz K., Makary M.S. (2022). Emerging curative-intent minimally-invasive therapies for hepatocellular carcinoma. World J. Hepatol..

[22] Criss C.R., Makary M.S. (2023). Recent Advances in Image-Guided Locoregional Therapies for Primary Liver Tumors. Biology.

[23] Gavriilidis P., Askari A., Azoulay D. (2017). Survival following redo hepatectomy vs radiofrequency ablation for recurrent hepatocellular carcinoma: A systematic review and meta-analysis. HPB.

[24] Li J.-K., Liu X.-H., Cui H., Xie X.-H. (2020). Radiofrequency ablation vs. surgical resection for resectable hepatocellular carcinoma: A systematic review and meta-analysis. Mol. Clin. Oncol..

[25] Park E.K., Kim H.J., Kim C.Y., Hur Y.H., Koh Y.S., Kim J.C., Kim H.J., Kim J.W., Cho C.K. (2014). A comparison between surgical resection and radiofrequency ablation in the treatment of hepatocellular carcinoma. Ann. Surg. Treat. Res..

[26] Kim G.-A., Shim J.H., Kim M.-J., Kim S.Y., Won H.J., Shin Y.M., Kim P.N., Kim K.-H., Lee S.-G., Lee H.C. (2016). Radiofrequency ablation as an alternative to hepatic resection for single small hepatocellular carcinomas. Br. J. Surg..

[27] Yousaf M.N., Ehsan H., Muneeb A., Wahab A., Sana M.K., Neupane K., Chaudhary F.S. (2021). Role of Radiofrequency Ablation in the Management of Unresectable Pancreatic Cancer. Front. Med..

[28] Testoni S.G.G., Healey A.J., Dietrich C.F., Arcidiacono P.G. (2020). Systematic review of endoscopy ultrasound-guided thermal ablation treatment for pancreatic cancer. Endosc. Ultrasound.

[29] Hare A.E., Makary M.S. (2022). Locoregional Approaches in Cholangiocarcinoma Treatment. Cancers.

[30] Owen M., Makary M.S., Beal E.W. (2023). Locoregional Therapy for Intrahepatic Cholangiocarcinoma. Cancers.

[31] Girelli R., Frigerio I., Salvia R., Barbi E., Tinazzi Martini P., Bassi C. (2010). Feasibility and safety of radiofrequency ablation for locally advanced pancreatic cancer. Br. J. Surg..

[32] D’Onofrio M., Crosara S., De Robertis R., Butturini G., Salvia R., Paiella S., Bassi C., Mucelli R.P. (2017). Percutaneous Radiofrequency Ablation of Unresectable Locally Advanced Pancreatic Cancer: Preliminary Results. Technol. Cancer Res. Treat..

[33] Mizandari M., Kumar J., Pai M., Chikovani T., Azrumelashvili T., Reccia I., Habib N. (2018). Interventional radiofrequency ablation: A promising therapeutic modality in the management of malignant biliary and pancreatic duct obstruction. J. Cancer.

[34] Gunn A.J., Gervais D.A. (2014). Percutaneous Ablation of the Small Renal Mass—Techniques and Outcomes. Semin. Interv. Radiol..

[35] Green H., Taylor A., Khoo V. (2023). Beyond the Knife in Renal Cell Carcinoma: A Systematic Review—To Ablate or Not to Ablate?. Cancers.

[36] Singh R.K., Gideon M., Rajendran R., Mathew G., Nair K. (2023). Advancing Treatment Frontiers: Radiofrequency Ablation for Small Renal Mass—Intermediate-Term Results. J. Kidney Cancer VHL.

[37] Bargellini I., Bozzi E., Cioni R., Parentini B., Bartolozzi C. (2011). Radiofrequency ablation of lung tumours. Insights Imaging.

[38] Zhao Q., Wang J., Fu Y., Hu B. (2023). Radiofrequency ablation for stage <IIB non-small cell lung cancer: Opportunities, challenges, and the road ahead. Thorac. Cancer.

[39] de Baère T., Aupérin A., Deschamps F., Chevallier P., Gaubert Y., Boige V., Fonck M., Escudier B., Palussiére J. (2015). Radiofrequency ablation is a valid treatment option for lung metastases: Experience in 566 patients with 1037 metastases. Ann. Oncol..

[40] Wang Y., Lu X., Wang Y., Li W., Li G., Zhou J. (2015). A prospective clinical trial of radiofrequency ablation for pulmonary metastases. Mol. Clin. Oncol..

[41] Matsui Y., Tomita K., Uka M., Umakoshi N., Kawabata T., Munetomo K., Nagata S., Iguchi T., Hiraki T. (2022). Up-to-date evidence on image-guided thermal ablation for metastatic lung tumors: A review. Jpn. J. Radiol..

[42] Wolf F.J., Grand D.J., Machan J.T., DiPetrillo T.A., Mayo-Smith W.W., Dupuy D.E. (2008). Microwave Ablation of Lung Malignancies: Effectiveness, CT Findings, and Safety in 50 Patients. Radiology.

[43] Wright A.S., Sampson L.A., Warner T.F., Mahvi D.M., Lee F.T. (2005). Radiofrequency versus Microwave Ablation in a Hepatic Porcine Model. Radiology.

[44] Strickland A.D., Clegg P.J., Cronin N.J., Swift B., Festing M., West K.P., Robertson G.S.M., Lloyd D.M. (2002). Experimental study of large-volume microwave ablation in the liver. Br. J. Surg..

[45] Liang P., Yu J., Lu M.-D., Dong B.-W., Yu X.-L., Zhou X.-D., Hu B., Xie M.-X., Cheng W., He W. (2013). Practice guidelines for ultrasound-guided percutaneous microwave ablation for hepatic malignancy. World J. Gastroenterol. (WJG).

[46] Reig M., Forner A., Rimola J., Ferrer-Fàbrega J., Burrel M., Garcia-Criado Á., Kelley R.K., Galle P.R., Mazzaferro V., Salem R. (2022). BCLC strategy for prognosis prediction and treatment recommendation: The 2022 update. J. Hepatol..

[47] Domini J., Makary M.S. (2023). Single-center analysis of percutaneous ablation in the treatment of hepatocellular carcinoma: Long-term outcomes of a 7-year experience. Abdom. Radiol..

[48] Radosevic A., Quesada R., Serlavos C., Sánchez J., Zugazaga A., Sierra A., Coll S., Busto M., Aguilar G., Flores D. (2022). Microwave versus radiofrequency ablation for the treatment of liver malignancies: A randomized controlled phase 2 trial. Sci. Rep..

[49] Forner A., Reig M., Bruix J. (2018). Hepatocellular carcinoma. Lancet.

[50] Zhou W., Arellano R.S. (2018). Thermal Ablation of T1c Renal Cell Carcinoma: A Comparative Assessment of Technical Performance, Procedural Outcome, and Safety of Microwave Ablation, Radiofrequency Ablation, and Cryoablation. J. Vasc. Interv. Radiol..

[51] Martin J., Athreya S. (2013). Meta-analysis of cryoablation versus microwave ablation for small renal masses: Is there a difference in outcome?. Diagn. Interv. Radiol..

[52] Zhou W., Herwald S.E., McCarthy C., Uppot R.N., Arellano R.S. (2019). Radiofrequency Ablation, Cryoablation, and Microwave Ablation for T1a Renal Cell Carcinoma: A Comparative Evaluation of Therapeutic and Renal Function Outcomes. J. Vasc. Interv. Radiol..

[53] Dong X., Li X., Yu J., Yu M., Yu X., Liang P. (2016). Complications of ultrasound-guided percutaneous microwave ablation of renal cell carcinoma. OncoTargets Ther..

[54] Jiang B., Mcclure M.A., Chen T., Chen S. (2018). Efficacy and safety of thermal ablation of lung malignancies: A Network meta-analysis. Ann. Thorac. Med..

[55] Chi J., Ding M., Shi Y., Wang T., Cui D., Tang X., Li P., Zhai B. (2018). Comparison study of computed tomography-guided radiofrequency and microwave ablation for pulmonary tumors: A retrospective, case-controlled observational study. Thorac. Cancer.

[56] Makary M.S., Khandpur U., Cloyd J.M., Mumtaz K., Dowell J.D. (2020). Locoregional Therapy Approaches for Hepatocellular Carcinoma: Recent Advances and Management Strategies. Cancers.

[57] Hiraki T., Gobara H., Fujiwara H., Ishii H., Tomita K., Uka M., Makimoto S., Kanazawa S. (2013). Lung cancer ablation: Complications. Semin. Interv. Radiol..

[58] Mal R., Domini J., Wadhwa V., Makary M.S. (2023). Thermal ablation for primary and metastatic lung tumors: Single-center analysis of peri-procedural and intermediate-term clinical outcomes. Clin. Imaging.

[59] Zheng A., Wang X., Yang X., Wang W., Huang G., Gai Y., Ye X. (2014). Major Complications After Lung Microwave Ablation: A Single-Center Experience on 204 Sessions. Ann. Thorac. Surg..

[60] Kashima M., Yamakado K., Takaki H., Kodama H., Yamada T., Uraki J., Nakatsuka A. (2011). Complications after 1000 lung radiofrequency ablation sessions in 420 patients: A single center’s experiences. Am. J. Roentgenol..

[61] Yuan Z., Wang Y., Zhang J., Zheng J., Li W. (2019). A Meta-Analysis of Clinical Outcomes After Radiofrequency Ablation and Microwave Ablation for Lung Cancer and Pulmonary Metastases. J. Am. Coll. Radiol..

[62] Carrafiello G., Ierardi A.M., Fontana F., Petrillo M., Floridi C., Lucchina N., Cuffari S., Dionigi G., Rotondo A., Fugazzola C. (2013). Microwave ablation of pancreatic head cancer: Safety and efficacy. J. Vasc. Interv. Radiol..

[63] Rombouts S.J.E., Vogel J.A., van Santvoort H.C., van Lienden K.P., van Hillegersberg R., Busch O.R.C., Besselink M.G.H., Molenaar I.Q. (2015). Systematic review of innovative ablative therapies for the treatment of locally advanced pancreatic cancer. Br. J. Surg..

[64] Ierardi A.M., Lucchina N., Bacuzzi A., Marco D.C., Bracchi E., Cocozza E., Dionigi G., Tsetis D., Floridi C., Carrafiello G. (2015). Percutaneous ablation therapies of inoperable pancreatic cancer: A systematic review. Ann. Gastroenterol. Q. Publ. Hell. Soc. Gastroenterol..

[65] Yamakado K. (2014). Image-guided ablation of adrenal lesions. Semin. Interv. Radiol..

[66] Donlon P., Dennedy M.C. (2021). Thermal ablation in adrenal disorders: A discussion of the technology, the clinical evidence and the future. Curr. Opin. Endocrinol. Diabetes Obes..

[67] Sarma A., Shyn P.B., Vivian M.A., Ng J.-M., Tuncali K., Lorch J.H., Zaheer S.N., Gordon M.S., Silverman S.G. (2015). Single-Session CT-Guided Percutaneous Microwave Ablation of Bilateral Adrenal Gland Hyperplasia Due to Ectopic ACTH Syndrome. Cardiovasc. Interv. Radiol..

[68] Fintelmann F.J., Tuncali K., Puchner S., Gervais D.A., Thabet A., Shyn P.B., Arellano R.S., Tatli S., Mueller P.R., Silverman S.G. (2016). Catecholamine Surge during Image-Guided Ablation of Adrenal Gland Metastases: Predictors, Consequences, and Recommendations for Management. J. Vasc. Interv. Radiol..

[69] Ren C., Liang P., Yu X.-L., Cheng Z.-G., Han Z.-Y., Yu J. (2016). Percutaneous microwave ablation of adrenal tumours under ultrasound guidance in 33 patients with 35 tumours: A single-centre experience. Int. J. Hyperth..

[70] Veltri A., Basile D., Calandri M., Bertaggia C., Volante M., Porpiglia F., Calabrese A., Puglisi S., Basile V., Terzolo M. (2020). Oligometastatic adrenocortical carcinoma: The role of image-guided thermal ablation. Eur. Radiol..

[71] Fingeret A.L. (2021). Contemporary Evaluation and Management of Parathyroid Carcinoma. JCO Oncol. Pract..

[72] Wei Y., Peng C.-Z., Wang S.-R., He J.-F., Peng L.-L., Zhao Z.-L., Cao X.-J., Li Y., Yu M.-A. (2021). Effectiveness and Safety of Thermal Ablation in the Treatment of Primary Hyperparathyroidism: A Multicenter Study. J. Clin. Endocrinol. Metab..

[73] Guo D.-M., Chen Z., Zhai Y.-X., Su H.-H. (2021). Comparison of radiofrequency ablation and microwave ablation for benign thyroid nodules: A systematic review and meta-analysis. Clin. Endocrinol..

[74] Choi Y., Jung S.-L. (2020). Efficacy and Safety of Thermal Ablation Techniques for the Treatment of Primary Papillary Thyroid Microcarcinoma: A Systematic Review and Meta-Analysis. Thyroid.

[75] Zhao Z.-L., Wang S.-R., Dong G., Liu Y., He J.-F., Shi L.-L., Guo J.-Q., Wang Z.-H., Cong Z.-B., Liu L.-H. (2024). Microwave Ablation versus Surgical Resection for US-detected Multifocal T1N0M0 Papillary Thyroid Carcinoma: A 10-Center Study. Radiology.

[76] Siskin G.P., Shlansky-Goldberg R.D., Goodwin S.C., Sterling K., Lipman J.C., Nosher J.L., Worthington-Kirsch R.L., Chambers T.P. (2006). A Prospective Multicenter Comparative Study between Myomectomy and Uterine Artery Embolization with Polyvinyl Alcohol Microspheres: Long-term Clinical Outcomes in Patients with Symptomatic Uterine Fibroids. J. Vasc. Interv. Radiol..

[77] Jin H., Lin W., Lu L., Cui M. (2021). Conventional thyroidectomy vs thyroid thermal ablation on postoperative quality of life and satisfaction for patients with benign thyroid nodules. Eur. J. Endocrinol..

[78] Gennaro N., Sconfienza L.M., Ambrogi F., Boveri S., Lanza E. (2019). Thermal ablation to relieve pain from metastatic bone disease: A systematic review. Skelet. Radiol..

[79] Wu M.-H., Xiao L.-F., Yan F.-F., Chen S.-L., Zhang C., Lei J., Deng Z.-M. (2019). Use of percutaneous microwave ablation for the treatment of bone tumors: A retrospective study of clinical outcomes in 47 patients. Cancer Imaging.

[80] Cazzato R.L., de Rubeis G., de Marini P., Dalili D., Koch G., Auloge P., Garnon J., Gangi A. (2021). Percutaneous microwave ablation of bone tumors: A systematic review. Eur. Radiol..

[81] Sprenkle P.C., Mirabile G., Durak E., Edelstein A., Gupta M., Hruby G.W., Okhunov Z., Landman J. (2010). The effect of argon gas pressure on ice ball size and rate of formation. J. Endourol..

[82] Baust J.G., Snyder K.K., Santucci K.L., Robilotto A.T., Van Buskirk R.G., Baust J.M. (2019). Cryoablation: Physical and molecular basis with putative immunological consequences. Int. J. Hyperth..

[83] Kwak K., Yu B., Lewandowski R.J., Kim D.-H. (2022). Recent progress in cryoablation cancer therapy and nanoparticles mediated cryoablation. Theranostics.

[84] Cha C., Lee F.T., Rikkers L.F., Niederhuber J.E., Nguyen B.T., Mahvi D.M. (2001). Rationale for the combination of cryoablation with surgical resection of hepatic tumors. J. Gastrointest. Surg..

[85] Sohn R.L., Carlin A.M., Steffes C., Tyburski J.G., Wilson R.F., Littrup P.J., Weaver D.W. (2003). The extent of cryosurgery increases the complication rate after hepatic cryoablation. Am. Surg..

[86] Niu L.-Z., Li J.-L., Xu K.-C. (2014). Percutaneous Cryoablation for Liver Cancer. J. Clin. Transl. Hepatol..

[87] Pearson A.S., Izzo F., Fleming R.Y., Ellis L.M., Delrio P., Roh M.S., Granchi J., Curley S.A. (1999). Intraoperative radiofrequency ablation or cryoablation for hepatic malignancies. Am. J. Surg..

[88] Yang Y., Wang C., Lu Y., Bai W., An L., Qu J., Gao X., Chen Y., Zhou L., Wu Y. (2012). Outcomes of ultrasound-guided percutaneous argon-helium cryoablation of hepatocellular carcinoma. J. Hepato-Biliary-Pancreat. Sci..

[89] Shafir M., Shapiro R., Sung M., Warner R., Sicular A., Klipfel A. (1996). Cryoablation of unresectable malignant liver tumors. Am. J. Surg..

[90] Conners D., Rilling W. (2011). Pleural tumor seeding following percutaneous cryoablation of hepatocellular carcinoma. Semin. Interv. Radiol..

[91] Fairchild A.H., Tatli S., Dunne R.M., Shyn P.B., Tuncali K., Silverman S.G. (2014). Percutaneous cryoablation of hepatic tumors adjacent to the gallbladder: Assessment of safety and effectiveness. J. Vasc. Interv. Radiol..

[92] Weight C.J., Lieser G., Larson B.T., Gao T., Lane B.R., Campbell S.C., Gill I.S., Novick A.C., Fergany A.F. (2010). Partial nephrectomy is associated with improved overall survival compared to radical nephrectomy in patients with unanticipated benign renal tumours. Eur. Urol..

[93] Atwell T.D., Schmit G.D., Boorjian S.A., Mandrekar J., Kurup A.N., Weisbrod A.J., Chow G.K., Leibovich B.C., Callstrom M.R., Patterson D.E. (2013). Percutaneous ablation of renal masses measuring 3.0 cm and smaller: Comparative local control and complications after radiofrequency ablation and cryoablation. Am. J. Roentgenol..

[94] Caputo P.A., Zargar H., Ramirez D., Andrade H.S., Akca O., Gao T., Kaouk J.H. (2017). Cryoablation versus Partial Nephrectomy for Clinical T1b Renal Tumors: A Matched Group Comparative Analysis. Eur. Urol..

[95] Tian Y., Qi X., Jiang X., Shang L., Xu K., Shao H. (2022). Cryoablation and immune synergistic effect for lung cancer: A review. Front. Immunol..

[96] Moore W., Talati R., Bhattacharji P., Bilfinger T. (2015). Five-year survival after cryoablation of stage I non-small cell lung cancer in medically inoperable patients. J. Vasc. Interv. Radiol..

[97] Lyons G.R., Askin G., Pua B.B. (2018). Clinical Outcomes after Pulmonary Cryoablation with the Use of a Triple Freeze Protocol. J. Vasc. Interv. Radiol..

[98] Pusceddu C., Sotgia B., Fele R.M., Melis L. (2013). CT-guided thin needles percutaneous cryoablation (PCA) in patients with primary and secondary lung tumors: A preliminary experience. Eur. J. Radiol..

[99] Gao W., Guo Z., Shu S., Xing W., Zhang W., Yang X. (2018). The application effect of percutaneous cryoablation for the stage IIIB/IV advanced non-small-cell lung cancer after the failure of chemoradiotherapy. Asian J. Surg..

[100] Yang W., An Y., Li Q., Liu C., Zhu B., Huang Q., Zhao M., Yang F., Feng H., Hu K. (2021). Co-ablation versus cryoablation for the treatment of stage III–IV non-small cell lung cancer: A prospective, noninferiority, randomized, controlled trial (RCT). Thorac. Cancer.

[101] Pfleiderer S.O.R., Freesmeyer M.G., Marx C., Kühne-Heid R., Schneider A., Kaiser W.A. (2002). Cryotherapy of breast cancer under ultrasound guidance: Initial results and limitations. Eur. Radiol..

[102] Fine R.E., Gilmore R.C., Dietz J.R., Boolbol S.K., Berry M.P., Han L.K., Kenler A.S., Sabel M., Tomkovich K.R., VanderWalde N.A. (2021). Cryoablation without Excision for Low-Risk Early-Stage Breast Cancer: 3-Year Interim Analysis of Ipsilateral Breast Tumor Recurrence in the ICE3 Trial. Ann. Surg. Oncol..

[103] Niu L., Zhou L., Xu K. (2012). Cryosurgery of breast cancer. Gland Surg..

[104] Simmons R.M., Ballman K.V., Cox C., Carp N., Sabol J., Hwang R.F., Attai D., Sabel M., Nathanson D., Kenler A. (2016). A Phase II Trial Exploring the Success of Cryoablation Therapy in the Treatment of Invasive Breast Carcinoma: Results from ACOSOG (Alliance) Z1072. Ann. Surg. Oncol..

[105] Vora B.M.K., Munk P.L., Somasundaram N., Ouellette H.A., Mallinson P.I., Sheikh A., Abdul Kadir H., Tan T.J., Yan Y.Y. (2021). Cryotherapy in extra-abdominal desmoid tumors: A systematic review and meta-analysis. PLoS ONE.

[106] Auloge P., Garnon J., Robinson J.M., Thenint M.-A., Koch G., Caudrelier J., Weiss J., Cazzato R.L., Kurtz J.E., Gangi A. (2021). Percutaneous cryoablation for advanced and refractory extra-abdominal desmoid tumors. Int. J. Clin. Oncol..

[107] Redifer Tremblay K., Lea W.B., Neilson J.C., King D.M., Tutton S.M. (2019). Percutaneous cryoablation for the treatment of extra-abdominal desmoid tumors. J. Surg. Oncol..

[108] Havez M., Lippa N., Al-Ammari S., Kind M., Stoeckle E., Italiano A., Gangi A., Hauger O., Cornelis F. (2014). Percutaneous image-guided cryoablation in inoperable extra-abdominal desmoid tumors: A study of tolerability and efficacy. Cardiovasc. Interv. Radiol..

[109] Schmitz J.J., Schmit G.D., Atwell T.D., Callstrom M.R., Kurup A.N., Weisbrod A.J., Morris J.M. (2016). Percutaneous Cryoablation of Extraabdominal Desmoid Tumors: A 10-Year Experience. Am. J. Roentgenol..

[110] Bouhamama A., Lame F., Mastier C., Cuinet M., Thibaut A., Beji H., Ricoeur A., Blay J.-Y., Pilleul F. (2020). Local Control and Analgesic Efficacy of Percutaneous Cryoablation for Desmoid Tumors. Cardiovasc. Interv. Radiol..

[111] Saltiel S., Bize P.E., Goetti P., Gallusser N., Cherix S., Denys A., Becce F., Tsoumakidou G. (2020). Cryoablation of Extra-Abdominal Desmoid Tumors: A Single-Center Experience with Literature Review. Diagnostics.

[112] Efrima B., Ovadia J., Drukman I., Khoury A., Rath E., Dadia S., Gortzak Y., Albagli A., Sternheim A., Segal O. (2021). Cryo-surgery for symptomatic extra-abdominal desmoids. A proof of concept study. J. Surg. Oncol..

[113] Eastham J.A., Auffenberg G.B., Barocas D.A., Chou R., Crispino T., Davis J.W., Eggener S., Horwitz E.M., Kane C.J., Kirkby E. (2022). Clinically Localized Prostate Cancer: AUA/ASTRO Guideline, Part I: Introduction, Risk Assessment, Staging, and Risk-Based Management. J. Urol..

[114] Gangi A., Tsoumakidou G., Abdelli O., Buy X., de Mathelin M., Jacqmin D., Lang H. (2012). Percutaneous MR-guided cryoablation of prostate cancer: Initial experience. Eur. Radiol..

[115] oodrum D.A., Kawashima A., Karnes R.J., Davis B.J., Frank I., Engen D.E., Gorny K.R., Felmlee J.P., Callstrom M.R., Mynderse L.A. (2013). Magnetic resonance imaging-guided cryoablation of recurrent prostate cancer after radical prostatectomy: Initial single institution experience. Urology.

[116] Bomers J.G.R., Yakar D., Overduin C.G., Sedelaar J.P.M., Vergunst H., Barentsz J.O., de Lange F., Fütterer J.J. (2013). MR Imaging–guided Focal Cryoablation in Patients with Recurrent Prostate Cancer. Radiology.

[117] De Marini P., Cazzato R.L., Garnon J., Tricard T., Koch G., Tsoumakidou G., Ramamurthy N., Lang H., Gangi A. (2019). Percutaneous MR-guided whole-gland prostate cancer cryoablation: Safety considerations and oncologic results in 30 consecutive patients. Br. J. Radiol..

[118] Kinsman K.A., White M.L., Mynderse L.A., Kawashima A., Rampton K., Gorny K.R., Atwell T.D., Felmlee J.P., Callstrom M.R., Woodrum D.A. (2018). Whole-Gland Prostate Cancer Cryoablation with Magnetic Resonance Imaging Guidance: One-Year Follow-Up. Cardiovasc. Interv. Radiol..

[119] Lomas D.J., Woodrum D.A., McLaren R.H., Gorny K.R., Felmlee J.P., Favazza C., Lu A., Mynderse L.A. (2020). Rectal wall saline displacement for improved margin during MRI-guided cryoablation of primary and recurrent prostate cancer. Abdom. Radiol..

[120] Chen C.-H., Pu Y.-S. (2014). Proactive rectal warming during total-gland prostate cryoablation. Cryobiology.

[121] de Castro Abreu A.L., Ma Y., Shoji S., Marien A., Leslie S., Gill I., Ukimura O. (2014). Denonvilliers’ space expansion by transperineal injection of hydrogel: Implications for focal therapy of prostate cancer. Int. J. Urol..

[122] Rubinsky B., Onik G., Mikus P. (2007). Irreversible electroporation: A new ablation modality—Clinical implications. Technol. Cancer Res. Treat..

[123] Hsiao C.-Y., Huang K.-W. (2017). Irreversible Electroporation: A Novel Ultrasound-guided Modality for Non-thermal Tumor Ablation. J. Med. Ultrasound.

[124] He C., Sun S., Zhang Y., Xie F., Li S. (2021). The role of irreversible electroporation in promoting M1 macrophage polarization via regulating the HMGB1-RAGE-MAPK axis in pancreatic cancer. OncoImmunology.

[125] He C., Huang X., Zhang Y., Lin X., Li S. (2020). T-cell activation and immune memory enhancement induced by irreversible electroporation in pancreatic cancer. Clin. Transl. Med..

[126] Babikr F., Wan J., Xu A., Wu Z., Ahmed S., Freywald A., Chibbar R., Wu Y., Moser M., Groot G. (2021). Distinct roles but cooperative effect of TLR3/9 agonists and PD-1 blockade in converting the immunotolerant microenvironment of irreversible electroporation-ablated tumors. Cell. Mol. Immunol..

[127] Scheffer H.J., Vroomen L.G.P.H., Nielsen K., van Tilborg A.A.J.M., Comans E.F.I., van Kuijk C., van der Meijs B.B., van den Bergh J., van den Tol P.M.P., Meijerink M.R. (2015). Colorectal liver metastatic disease: Efficacy of irreversible electroporation—A single-arm phase II clinical trial (COLDFIRE-2 trial). BMC Cancer.

[128] Ruarus A.H., Vroomen L.G.P.H., Puijk R.S., Scheffer H.J., Zonderhuis B.M., Kazemier G., van den Tol M.P., Berger F.H., Meijerink M.R. (2018). Irreversible Electroporation in Hepatopancreaticobiliary Tumours. Can. Assoc. Radiol. J..

[129] Kluger M.D., Epelboym I., Schrope B.A., Mahendraraj K., Hecht E.M., Susman J., Weintraub J.L., Chabot J.A. (2016). Single-Institution Experience with Irreversible Electroporation for T4 Pancreatic Cancer: First 50 Patients. Ann. Surg. Oncol..

[130] Martin R.C.G.I., Kwon D., Chalikonda S., Sellers M., Kotz E., Scoggins C., McMasters K.M., Watkins K. (2015). Treatment of 200 Locally Advanced (Stage III) Pancreatic Adenocarcinoma Patients with Irreversible Electroporation: Safety and Efficacy. Ann. Surg..

[131] Scheffer H.J., Vroomen L.G.P.H., de Jong M.C., Melenhorst M.C.A.M., Zonderhuis B.M., Daams F., Vogel J.A., Besselink M.G.H., van Kuijk C., Witvliet J. (2017). Ablation of Locally Advanced Pancreatic Cancer with Percutaneous Irreversible Electroporation: Results of the Phase I/II PANFIRE Study. Radiology.

[132] Lum M.A., Shah S.B., Durack J.C., Nikolovski I. (2019). Imaging of Small Renal Masses before and after Thermal Ablation. RadioGraphics.

[133] Pech M., Janitzky A., Wendler J.J., Strang C., Blaschke S., Dudeck O., Ricke J., Liehr U.-B. (2011). Irreversible Electroporation of Renal Cell Carcinoma: A First-in-Man Phase I Clinical Study. Cardiovasc. Interv. Radiol..

[134] Canvasser N.E., Sorokin I., Lay A.H., Morgan M.S.C., Ozayar A., Trimmer C., Cadeddu J.A. (2017). Irreversible electroporation of small renal masses: Suboptimal oncologic efficacy in an early series. World J. Urol..

[135] Tasu J.-P., Tougeron D., Rols M.-P. (2022). Irreversible electroporation and electrochemotherapy in oncology: State of the art. Diagn. Interv. Imaging.

[136] Onik G., Mikus P., Rubinsky B. (2007). Irreversible Electroporation: Implications for Prostate Ablation. Technol. Cancer Res. Treat..

[137] Neal II R.E., Millar J.L., Kavnoudias H., Royce P., Rosenfeldt F., Pham A., Smith R., Davalos R.V., Thomson K.R. (2014). In vivo characterization and numerical simulation of prostate properties for non-thermal irreversible electroporation ablation. Prostate.

[138] Blazevski A., Scheltema M.J., Yuen B., Masand N., Nguyen T.V., Delprado W., Shnier R., Haynes A.-M., Cusick T., Thompson J. (2020). Oncological and Quality-of-life Outcomes Following Focal Irreversible Electroporation as Primary Treatment for Localised Prostate Cancer: A Biopsy-monitored Prospective Cohort. Eur. Urol. Oncol..

[139] Blazevski A., Amin A., Scheltema M.J., Balakrishnan A., Haynes A.-M., Barreto D., Cusick T., Thompson J., Stricker P.D. (2021). Focal ablation of apical prostate cancer lesions with irreversible electroporation (IRE). World J. Urol..

[140] Wang H., Xue W., Yan W., Yin L., Dong B., He B., Yu Y., Shi W., Zhou Z., Lin H. (2022). Extended Focal Ablation of Localized Prostate Cancer with High-Frequency Irreversible Electroporation: A Nonrandomized Controlled Trial. JAMA Surg..

[141] Kalapara A.A., Ballok Z.E., Ramdave S., O’Sullivan R., Ryan A., Konety B., Grummet J.P., Frydenberg M. (2022). Combined Utility of 68Ga-Prostate-specific Membrane Antigen Positron Emission Tomography/Computed Tomography and Multiparametric Magnetic Resonance Imaging in Predicting Prostate Biopsy Pathology. Eur. Urol. Oncol..

[142] Kalapara A.A., Verbeek J.F.M., Nieboer D., Fahey M., Gnanapragasam V., Van Hemelrijck M., Lee L.S., Bangma C.H., Steyerberg E.W., Harkin T. (2020). Adherence to Active Surveillance Protocols for Low-risk Prostate Cancer: Results of the Movember Foundation’s Global Action Plan Prostate Cancer Active Surveillance Initiative. Eur. Urol. Oncol..

[143] Izadifar Z., Izadifar Z., Chapman D., Babyn P. (2020). An Introduction to High Intensity Focused Ultrasound: Systematic Review on Principles, Devices, and Clinical Applications. J. Clin. Med..

[144] Zhou Y.-F. (2011). High intensity focused ultrasound in clinical tumor ablation. World J. Clin. Oncol..

[145] Dewey W.C. (2009). Arrhenius relationships from the molecule and cell to the clinic. Int. J. Hyperth..

[146] Lagneaux L., de Meulenaer E.C., Delforge A., Dejeneffe M., Massy M., Moerman C., Hannecart B., Canivet Y., Lepeltier M.F., Bron D. (2002). Ultrasonic low-energy treatment: A novel approach to induce apoptosis in human leukemic cells. Exp. Hematol..

[147] Salgaonkar V.A., Diederich C.J. (2015). Catheter-based ultrasound technology for image-guided thermal therapy: Current technology and applications. Int. J. Hyperth..

[148] Wu F., Chen W.Z., Bai J., Zou J.Z., Wang Z.L., Zhu H., Wang Z.B. (2001). Pathological changes in human malignant carcinoma treated with high-intensity focused ultrasound. Ultrasound Med. Biol..

[149] Wu F., Wang Z.-B., Chen W.-Z., Wang W., Gui Y., Zhang M., Zheng G., Zhou Y., Xu G., Li M. (2004). Extracorporeal high intensity focused ultrasound ablation in the treatment of 1038 patients with solid carcinomas in China: An overview. Ultrason. Sonochem..

[150] Li C.-X., Xu G.-L., Jiang Z.-Y., Li J.-J., Luo G.-Y., Shan H.-B., Zhang R., Li Y. (2004). Analysis of clinical effect of high-intensity focused ultrasound on liver cancer. World J. Gastroenterol..

[151] Wu F., Wang Z.-B., Chen W.-Z., Zou J.-Z., Bai J., Zhu H., Li K.-Q., Jin C.-B., Xie F.-L., Su H.-B. (2005). Advanced hepatocellular carcinoma: Treatment with high-intensity focused ultrasound ablation combined with transcatheter arterial embolization. Radiology.

[152] Wu F., Wang Z.-B., Cao Y.-D., Zhu X.-Q., Zhu H., Chen W.-Z., Zou J.-Z. (2007). “Wide local ablation” of localized breast cancer using high intensity focused ultrasound. J. Surg. Oncol..

[153] Furusawa H., Namba K., Thomsen S., Akiyama F., Bendet A., Tanaka C., Yasuda Y., Nakahara H. (2006). Magnetic resonance–guided focused ultrasound surgery of breast cancer: Reliability and effectiveness. J. Am. Coll. Surg..

[154] Wu F., Wang Z.-B., Zhu H., Chen W.-Z., Zou J.-Z., Bai J., Li K.-Q., Jin C.-B., Xie F.-L., Su H.-B. (2005). Extracorporeal high intensity focused ultrasound treatment for patients with breast cancer. Breast Cancer Res. Treat..

[155] Wu F., Wang Z.-B., Cao Y.-D., Chen W.-Z., Bai J., Zou J.-Z., Zhu H. (2003). A randomised clinical trial of high-intensity focused ultrasound ablation for the treatment of patients with localised breast cancer. Br. J. Cancer.

[156] Gianfelice D., Khiat A., Amara M., Belblidia A., Boulanger Y. (2003). MR imaging–guided focused US ablation of breast cancer: Histopathologic assessment of effectiveness—initial experience. Radiology.

[157] Furusawa H., Namba K., Nakahara H., Tanaka C., Yasuda Y., Hirabara E., Imahariyama M., Komaki K. (2007). The evolving non-surgical ablation of breast cancer: MR guided focused ultrasound (MRgFUS). Breast Cancer.

[158] Ahmed H.U., Zacharakis E., Dudderidge T., Armitage J.N., Scott R., Calleary J., Illing R., Kirkham A., Freeman A., Ogden C. (2009). High-intensity-focused ultrasound in the treatment of primary prostate cancer: The first UK series. Br. J. Cancer.

[159] Chaussy C., Thüroff S. (2003). The status of high-intensity focused ultrasound in the treatment of localized prostate cancer and the impact of a combined resection. Curr. Urol. Rep..

[160] Beerlage H.P., Thüroff S., Debruyne F.M., Chaussy C., de la Rosette J.J. (1999). Transrectal high-intensity focused ultrasound using the Ablatherm device in the treatment of localized prostate carcinoma. Urology.

[161] Chaussy C., Thüroff S. (2000). High-intensity focused ultrasound in prostate cancer: Results after 3 years. Mol. Urol..

[162] Ripert T., Azémar M.-D., Ménard J., Bayoud Y., Messaoudi R., Duval F., Staerman F. (2010). Transrectal high-intensity focused ultrasound (HIFU) treatment of localized prostate cancer: Review of technical incidents and morbidity after 5 years of use. Prostate Cancer Prostatic Dis..

[163] Hatiboglu G., Popeneciu I.V., Deppert M., Nyarangi-Dix J., Hadaschik B., Hohenfellner M., Teber D., Pahernik S. (2017). Quality of life and functional outcome after infravesical desobstruction and HIFU treatment for localized prostate cancer. BMC Urol..

[164] Sanda M.G., Cadeddu J.A., Kirkby E., Chen R.C., Crispino T., Fontanarosa J., Freedland S.J., Greene K., Klotz L.H., Makarov D.V. (2018). Clinically Localized Prostate Cancer: AUA/ASTRO/SUO Guideline. Part I: Risk Stratification, Shared Decision Making, and Care Options. J. Urol..

[165] Abdelsalam M.E., Ahrar K. (2020). Ablation of Small Renal Masses. Tech. Vasc. Interv. Radiol..

[166] Klingler H.C., Susani M., Seip R., Mauermann J., Sanghvi N., Marberger M.J. (2008). A novel approach to energy ablative therapy of small renal tumours: Laparoscopic high-intensity focused ultrasound. Eur. Urol..

[167] Illing R.O., Kennedy J.E., Wu F., ter Haar G.R., Protheroe A.S., Friend P.J., Gleeson F.V., Cranston D.W., Phillips R.R., Middleton M.R. (2005). The safety and feasibility of extracorporeal high-intensity focused ultrasound (HIFU) for the treatment of liver and kidney tumours in a Western population. Br. J. Cancer.

[168] Köhrmann K.U., Michel M.S., Gaa J., Marlinghaus E., Alken P. (2002). High intensity focused ultrasound as noninvasive therapy for multilocal renal cell carcinoma: Case study and review of the literature. J. Urol..

[169] Napier K.J., Scheerer M., Misra S. (2014). Esophageal cancer: A Review of epidemiology, pathogenesis, staging workup and treatment modalities. World J. Gastrointest. Oncol..

[170] Melodelima D., Prat F., Fritsch J., Theillere Y., Cathignol D. (2008). Treatment of esophageal tumors using high intensity intraluminal ultrasound: First clinical results. J. Transl. Med..

[171] Jung S.E., Cho S.H., Jang J.H., Han J.-Y. (2011). High-intensity focused ultrasound ablation in hepatic and pancreatic cancer: Complications. Abdom. Imaging.

[172] Xiong L.L., Hwang J.H., Huang X.B., Yao S.S., He C.J., Ge X.H., Ge H.Y., Wang X.F. (2009). Early clinical experience using high intensity focused ultrasound for palliation of inoperable pancreatic cancer. JOP J. Pancreas.

[173] Zhang F.-Q., Li L., Huang P.-C., Xia F.-F., Zhu L., Cao C. (2021). Stent Insertion with High Intensity-Focused Ultrasound Ablation for Biliary Obstruction Caused by Pancreatic Carcinoma: A Randomized Controlled Trial. Surg. Laparosc. Endosc. Percutan. Tech..

[174] Zhao H., Yang G., Wang D., Yu X., Zhang Y., Zhu J., Ji Y., Zhong B., Zhao W., Yang Z. (2010). Concurrent gemcitabine and high-intensity focused ultrasound therapy in patients with locally advanced pancreatic cancer. Anti-Cancer Drugs.

[175] Lipsman N., Mainprize T.G., Schwartz M.L., Hynynen K., Lozano A.M. (2014). Intracranial applications of magnetic resonance-guided focused ultrasound. Neurotherapeutics.

[176] McDannold N., Clement G.T., Black P., Jolesz F., Hynynen K. (2010). Transcranial magnetic resonance imaging-guided focused ultrasound surgery of brain tumors: Initial findings in 3 patients. Neurosurgery.

[177] Cohen-Inbar O., Xu Z., Sheehan J.P. (2016). Focused ultrasound-aided immunomodulation in glioblastoma multiforme: A therapeutic concept. J. Ther. Ultrasound.

[178] Hynynen K., Chung A.H., Colucci V., Jolesz F.A. (1996). Potential adverse effects of high-intensity focused ultrasound exposure on blood vessels in vivo. Ultrasound Med. Biol..

[179] Li C., Zhang W., Fan W., Huang J., Zhang F., Wu P. (2010). Noninvasive treatment of malignant bone tumors using high-intensity focused ultrasound. Cancer.

[180] Liberman B., Gianfelice D., Inbar Y., Beck A., Rabin T., Shabshin N., Chander G., Hengst S., Pfeffer R., Chechick A. (2009). Pain palliation in patients with bone metastases using MR-guided focused ultrasound surgery: A multicenter study. Ann. Surg. Oncol..

[181] Xu Z., Hall T.L., Vlaisavljevich E., Lee F.T. (2021). Histotripsy: The first noninvasive, non-ionizing, non-thermal ablation technique based on ultrasound. Int. J. Hyperth..

[182] HistoSonics, Inc. (2024). The HistoSonics Edison System for Treatment of Primary Solid Renal Tumors Using Histotripsy (#HOPE4KIDNEY US).

